# Seeking order amidst chaos: a systematic review of classification systems for causes of stillbirth and neonatal death, 2009–2014

**DOI:** 10.1186/s12884-016-1071-0

**Published:** 2016-10-05

**Authors:** Susannah Hopkins Leisher, Zheyi Teoh, Hanna Reinebrant, Emma Allanson, Hannah Blencowe, Jan Jaap Erwich, J. Frederik Frøen, Jason Gardosi, Sanne Gordijn, A. Metin Gülmezoglu, Alexander E. P. Heazell, Fleurisca Korteweg, Joy Lawn, Elizabeth M. McClure, Robert Pattinson, Gordon C. S. Smith, Ӧzge Tunçalp, Aleena M. Wojcieszek, Vicki Flenady

**Affiliations:** 1 Mater Research Institute, The University of Queensland (MRI-UQ), Brisbane, Australia; 2 International Stillbirth Alliance, Millburn, USA; 3 Department of Reproductive Health and Research including UNDP/UNFPA/UNICEF/WHO/World Bank Special Programme of Research, Development and Research Training in Human Reproduction (HRP), World Health Organization, Geneva, Switzerland; 4 School of Women’s and Infants’ Health, Faculty of Medicine, Dentistry and Health Sciences, University of Western Australia, Perth, Australia; 5 London School of Hygiene & Tropical Medicine, London, UK; 6 The University of Groningen, University Medical Center Groningen, Groningen, The Netherlands; 7 Department of International Public Health, Norwegian Institute of Public Health, Oslo, Norway; 8 Center for Intervention Science for Maternal and Child Health, University of Bergen, Bergen, Norway; 9 Perinatal Institute, Birmingham, UK; 10 Maternal and Fetal Health Research Centre, University of Manchester, Manchester, UK; 11 St. Mary’s Hospital, Central Manchester University Hospitals NHS Foundation Trust, Manchester Academic Health Science Centre, Manchester, UK; 12 Department of Obstetrics and Gynaecology, Martini Hospital, Groningen, The Netherlands; 13 Research Triangle Institute, North Carolina, USA; 14 South Africa Medical Research Council Maternal and Infant Health Care Strategies Unit, University of Pretoria, Pretoria, South Africa; 15 NIHR Biomedical Research Centre & Department of Obstetrics & Gynaecology, Cambridge University, Cambridge, UK

**Keywords:** Stillbirth, Neonatal death, Perinatal death, Classification system, Classification, Cause of death

## Abstract

**Background:**

Each year, about 5.3 million babies die in the perinatal period. Understanding of causes of death is critical for prevention, yet there is no globally acceptable classification system. Instead, many disparate systems have been developed and used. We aimed to identify all systems used or created between 2009 and 2014, with their key features, including extent of alignment with the International Classification of Diseases (ICD) and variation in features by region, to inform the World Health Organization’s development of a new global approach to classifying perinatal deaths.

**Methods:**

A systematic literature review (CINAHL, EMBASE, Medline, Global Health, and PubMed) identified published and unpublished studies and national reports describing new classification systems or modifications of existing systems for causes of perinatal death, or that used or tested such systems, between 2009 and 2014. Studies reporting ICD use only were excluded. Data were independently double-extracted (except from non-English publications). Subgroup analyses explored variation by extent and region.

**Results:**

Eighty-one systems were identified as new, modifications of existing systems, or having been used between 2009 and 2014, with an average of ten systems created/modified each year. Systems had widely varying characteristics: (i) comprehensiveness (40 systems classified both stillbirths and neonatal deaths); (ii) extent of use (systems were created in 28 countries and used in 40; 17 were created for national use; 27 were widely used); (iii) accessibility (three systems available in e-format); (iv) underlying cause of death (64 systems required a single cause of death); (v) reliability (10 systems tested for reliability, with overall Kappa scores ranging from .35–.93); and (vi) ICD alignment (17 systems used ICD codes). Regional databases were not searched, so system numbers may be underestimated. Some non-differential misclassification of systems was possible.

**Conclusions:**

The plethora of systems in use, and continuing system development, hamper international efforts to improve understanding of causes of death. Recognition of the features of currently used systems, combined with a better understanding of the drivers of continued system creation, may help the development of a truly effective global system.

**Electronic supplementary material:**

The online version of this article (doi:10.1186/s12884-016-1071-0) contains supplementary material, which is available to authorized users.

## Background

Each year, approximately 2.6 million babies are stillborn in their third trimester, about half of these during labour (intrapartum stillbirths). Another 2.7 million are born alive only to die within their first month [1, 2]. With 5.3 million deaths a year, perinatal death is a tragedy on a par with under-5 deaths (5.9 million [1]), and has far-reaching effects for bereaved families, caregivers, and ultimately society at large [3]. Understanding the causes of stillbirths and neonatal deaths is critical for prevention. Systems that classify causes are thus indispensable tools for researchers, policy makers and caregivers working to reduce the numbers of these deaths.

Classification systems for causes of stillbirth and neonatal death are roughly a century old. The first systems originated in Scotland to classify causes based on clinically observable factors [4]. In 1941, Baird developed what has become one of the most widely used classification systems, referred to as the “Aberdeen,” which aimed to reduce the percentage of unexplained deaths [5]. Early modifications to the Aberdeen added categories, provided definitions to increase consistency of interpretation, and incorporated World Health Organization (WHO) definitions for low birthweight. A new family of systems with more focus on autopsy results was established in 1956 by Bound [6]. This system was modified for use by the British Perinatal Mortality Survey, with several other subsequent modifications [4]. In 1980, Wigglesworth launched a third family using categories that were simple to apply, clinically actionable, and did not require autopsy [7]. The Wigglesworth system has been used and adapted widely [8]. Numerous other types of systems have been developed to classify causes of both stillbirth and neonatal deaths, for instance systems based on placental pathology [9], distinguishing between immediate and underlying causes [10, 11], combining autopsy results with clinical data [12], incorporating deaths both before birth and through infancy [13], and exploring preventability rather than causality [14].

There is a recognized need to rationalize approaches to cause-of-death classification. The Lancet’s 2011 stillbirth series called for the creation of a “universal classification system” for causes of stillbirth [15, 16], and the United Nations-endorsed *Every Newborn Action Plan* (2014) identified cause of death as a key gap in the available data, proposing registration of all stillbirths and neonatal deaths together with identification of cause of death as one of the plan’s global indicators [17].

While it is improving, under-reporting of perinatal deaths (particularly stillbirths) in some of the highest-burden regions is still problematic [2]. In recognition of the need to increase accurate data capture and reporting, the WHO is currently developing a new approach to perinatal death classification for global use, the “WHO Application of the ICD-10 to perinatal deaths” (ICD-Perinatal Mortality or ICD-PM) [18]. Having a separate ICD module for perinatal deaths which incorporates both maternal and fetal/neonatal conditions, in recognition of the mother-baby dyad, is intended to increase reporting of perinatal deaths globally, as well as improving data accuracy.

Several reviews of classification systems for causes of stillbirth and neonatal death have been undertaken, yet all have been limited by one or more factors, including type of death (most were stillbirth-only) and scope (time period, languages included, etc.) [8, 19–21]. The aim of this systematic review was to gain an understanding of classification systems that have been developed or used recently in order to inform the ICD-PM and plans for its implementation. Specific objectives were to:

1. identify classification systems for causes of stillbirth and neonatal death which have been developed as new systems, modified from existing systems, or used between 2009 and 2014;
2. describe the characteristics of these systems, including any reliability testing performed;
3. describe the alignment of these systems with the ICD; and
4. examine variation in Objectives 1–3 according to country economic region as defined by the World Bank [22].

This paper presents findings from the first of a two-part study. The second part presents an assessment of alignment of the systems identified and reported on in the present paper with expert-identified characteristics for a globally acceptable system, and is also reported in the BMC Ending Preventable Stillbirths series [23].

## Methods

A systematic literature review was undertaken using principles of the Cochrane Collaboration [24], including a comprehensive search, and study selection and data extraction independently undertaken by two authors. The senior author resolved differences; otherwise, system developers who are co-authors were excluded from selection of studies, data extraction and analysis. See Additional file 1 for the PRISMA checklist. 

### Inclusion criteria

We included published and unpublished studies reporting classification systems for stillbirths (SB) and/or neonatal deaths (NND) that were created, modified, and/or used between 2009 and 2014. The inclusion criteria were:

1. All publications between 2009 and 2014 that:
  - described at least one new and/or modified classification system for causes of SB and/or NND or
  - reported data on causes of SB and/or NND using any classification system, regardless of when that system was created or modified.
2. For any systems that were found to be used between 2009 and 2014, as in (1-b) above, we also included the publication that was provided as the reference for that system, regardless of whether it was published in 2009–2014 or earlier.
3. All publications between 2009 and 2014 that reported on reliability testing of any systems included via (1) and (2) above.
4. The most recent publication between 2009 and 2014 in English that described a national system.

The original search period was the ten years from 2004–2013; this was halved (to 2009–2013) due to resource limitations, and because data extraction extended into 2014, a sixth year was added to the search period. Systems classifying SB were included regardless of the gestation at which SB was defined in included publications. Systems classifying both early (0–7 days) and late (8–28 days) NND were included, as well as systems classifying perinatal deaths without separation into SB and NND.

The rationale for including modifications of original systems was twofold. First, even slight modification of a system may render its data less compatible with other systems, and second, modification may reflect users’ perceptions of the inadequacy of available systems.

**Fig. 1:**
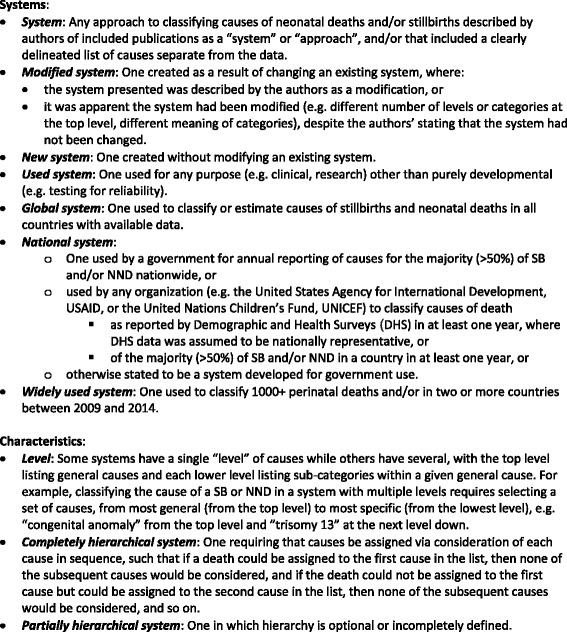
Definitions of terms used

See Fig. 1 for definitions of terms used.

### Exclusion criteria

Systems developed for specific populations (e.g., unexplained SB at term, low birthweight babies) were excluded. Systems for which data on SB, NND, and/or perinatal deaths could not be separated from data on deaths before or after the perinatal period (e.g., miscarriages, late infant deaths) were excluded. Because our ultimate aim was to inform development and optimize successful uptake of a new global system, we needed to gain an understanding of the context of systems development beyond the ICD. This meant our focus was on understanding the features of systems developed by users, and thus which reflected their needs. Hence, papers describing use of only the ICD were also excluded.

### Search strategy and study selection

**Fig. 2:**
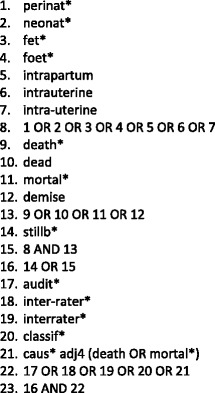
Search string

Five electronic databases (CINAHL, EMBASE, Global Health, MEDLINE, and PubMed) were searched for the period January 1, 2009, to December 31, 2014, with no language limits (see Fig. 2 for search string). In addition, an English-language search was carried out to identify all national systems in use. Searches were supplemented by contacting expert informants.

Every English-language paper was independently screened for inclusion by two authors in two stages—abstract review and full text review—with final decisions made by the senior author in the event of disagreement (see Additional file 2 for decision tree on inclusion/exclusion). Screening of non-English papers at the abstract stage was performed in the same way, but full-text review was done by one of three researchers (depending on language) with guidance by the first author.

### Data collection

A data collection tool was purpose-built and pilot tested for data extraction of 48 variables (see Additional file 3), including:

1. 21 variables to describe basic system features such as year of publication, whether systems were new or modified, whether authors intended to create or modify systems or merely to use existing systems, and authors’ descriptions of reasons for system creation;
2. 26 variables to enable assessment of alignment with expert-identified characteristics for a globally acceptable system (see [23]), including variables for:
  - Comprehensiveness (e.g. whether both SB and NND were included, and whether associated factors were recorded);
  - Extent of use (e.g. regions of origin and use, number of deaths classified, and whether national or not);
  - Accessibility and relevance (e.g. whether available in e-format and multiple languages and whether guidance for accessing data was provided; also, although verbal autopsy is a data collection tool, we recorded whether systems had been used with verbal autopsy as one proxy for a system’s relevance in low-resource settings);
  - Identification of underlying causes (e.g. maximum % “other” recorded by any use of the systems in included papers, number of causes in top “level”, number of levels, and whether fully, partially, or not hierarchical; see Fig. 1 for definitions of terms);
  - Reliability (including whether rules for assigning cause of death and definitions of causes were provided);
3. One variable to record whether ICD codes were used. This variable was included in data extraction as it was known to be important for development of the ICD-PM.

Data for variables relating to basic system features were taken both from publications that introduced new or modified systems between 2009 and 2014, and from older publications if they had been cited as the source of a system used within 2009–2014, regardless of year of publication. Data relating to the use of the systems (included in #2 above), for instance number of deaths classified, countries in which used, and percent of deaths classified as “other”, were taken from publications within 2009–2014 that described use of these systems. Therefore, a system described in a publication from 1970 would be included only if it had been used at least once in a publication between 2009 and 2014; all data relating to use of this system would be taken only from the latter publication, while all data relating to the system’s basic features would be taken from the former publication.

Data from English publications were independently double-extracted; any disagreements were resolved by the senior author. Data from non-English publications were extracted by the same researchers who had performed full-text review of these publications, with the guidance of the first author. Where multiple systems were included in a single publication, each was extracted separately.

### Data management and analysis

Data were entered into Microsoft Excel 2013. Coding was independently checked by a second researcher, and then imported to Stata/IC 12.1 for analysis of frequency distributions. Subgroup analyses were performed to explore differences in frequencies according to extent of use (whether widely used, region in which used, and use in highest-burden countries). A sensitivity analysis was carried out to explore the implications of cut-offs for identification of widely used systems (see Additional file 4 for method).

For a copy of the study protocol, please contact the author.

## Results

### Search results

**Fig. 3:**
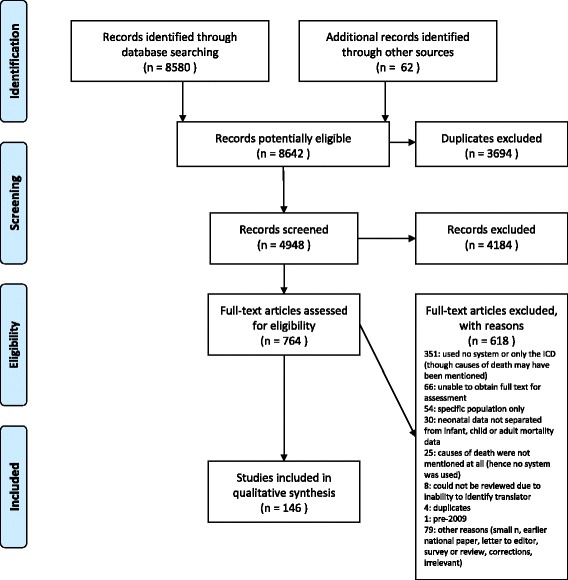
Classification systems for causes of stillbirths and neonatal deaths, 2009–2014: PRISMA flow diagram

In total, 4,948 publications were screened for eligibility, 764 were assessed for eligibility, and 146 were included (Fig. 3). Some included publications met more than one inclusion criterion (e.g., included both a description of a new system and use of an existing system) (see Additional file 5 for all included publications with reasons for inclusion). Of included publications, 11 presented systems that were newly created, 40 presented systems that were modified, 81 presented system use (including 17 systems that had been created prior to 2009), and 15 presented the results of reliability testing for one or more included systems. 120 non-English publications in 16 languages were screened via English abstracts, with publications in eight non-English languages identified for full-text review. Eight publications in Persian were excluded due to the inability to identify a translator. See Fig. 3 for a summary of reasons for exclusion.

### System creation and use

#### Number and year of creation of systems

A total of 81 systems were created, modified, and/or used between 2009 and 2014.^1^ The oldest system in use was Wigglesworth 1980, while two systems created in 2014 had no published record of use (McClure 2014-Global Network and Gardosi 2014-MAIN). An average of 10 systems were created or modified annually between 2009 and 2014 (see Additional file 6).

#### New and modified systems compared to author intent

The majority of systems (n = 59, 73 %) were modifications of existing systems. Of the 14 systems that we defined as new, 10 were also intended by their authors as new systems. Of the remaining four, two were intended as new approaches rather than new systems, one was intended as a use of an existing system, and one was not intended as a use or creation of any system. Just 22 of the 59 systems defined by us as modifications were intended by their authors as such. A further 27 were intended as uses of existing systems, with the modifications that we found going unmentioned by the authors; five were intended as new systems, and the remaining five had other intents. We were unable to determine whether eight systems were new or modified; of these, six were intended as uses of existing systems, while author intent for the remaining two could not be determined (see Table 1 and Additional file 5).

**Table 1:** Selected characteristics of classification systems for causes of stillbirth and neonatal death, 2009–2014

Characteristic	All systems, n (%)	Systems used in HIC only, n (%)	Systems used in LMIC only, n (%)
For systems including any type of death (SB, NND, or both)	81	36	32
Type of system			
New	14 (17)	6 (17)	4 (13)
Modified	59 (73)	28 (78)	23 (72)
Unknown	8 (10)	2 (6)	5 (16)
Uses ICD codes			
Yes	17 (21)	3 (8)	8 (25)
No	62 (77)	33 (92)	23 (72)
Unclear	2 (3)	0 (0)	1 (3)
Includes definitions for all causes of death			
Yes	23 (28)	9 (25)	11 (34)
No	35 (43)	14 (39)	16 (50)
Some causes only	23 (28)	13 (36)	5 (16)
Includes a description of how COD are to be assigned			
Yes	33 (41)	16 (44)	9 (28)
No	47 (58)	20 (56)	23 (72)
Unclear	1 (1)	0 (0)	0 (0)
Number of deaths classified using this system			
Not used	5 (6)	0 (0)	0 (0)
< 500	44 (54)	17 (47)	26 (81)
500-999	9 (11)	7 (19)	2 (6)
1000+	23 (28)	12 (33)	4 (13)
Includes guidance on how potential users might access data from the system			
Yes	8 (10)	5 (14)	2 (6)
No	66 (82)	24 (67)	30 (94)
Unclear	7 (9)	7 (19)	0 (0)
Available in e-format	3 (4)	2 (6)	1 (3)
Available in more than 1 language	1 (1)	1 (3)	0 (0)
Type of death classified			
Both SB and NND	40 (49)	20 (56)	14 (44)
NND only	26 (32)	7 (19)	14 (44)
SB only	15 (19)	9 (25)	4 (13)
Number of countries in which used			
0	5 (6)	0 (0)	0 (0)
1	60 (74)	30 (83)	30 (94)
2+	13 (16)	6 (17)	2 (6)
Used to report global data	3 (4)	0 (0)	0 (0)
Tested for reliability			
Yes	10 (12)	4 (11)	3 (9)
No	68 (84)	31 (86)	28 (88)
Unclear	3 (4)	1 (3)	1 (3)
Hierarchical			
Yes	18 (22)	4 (11)	10 (31)
No	53 (65)	27 (75)	20 (63)
Partially	7 (9)	4 (11)	0 (0)
Unclear	3 (4)	1 (3)	2 (6)
Requires that a single cause of death be recorded			
Yes	64 (79)	29 (81)	23 (72)
No	12 (15)	6 (17)	5 (16)
Unclear	5 (6)	1 (3)	4 (13)
List of causes does not include FGR, IUGR or SGA			
Yes	65 (80)	27 (75)	28 (88)
No	16 (20)	9 (25)	4 (13)
Allows associated factors to be recorded			
Yes	23 (28)	13 (36)	6 (19)
No	57 (70)	23 (64)	25 (78)
Unclear	1 (1)	0 (0)	1 (3)
Number of categories in top level			
≤ 10	67 (83)	26 (72)	28 (88)
> 10	14 (17)	10 (28)	4 (13)
Number of levels			
> 1	44 (54)	21 (58)	17 (53)
1	35 (43)	15 (42)	14 (44)
Unclear	2 (3)	0 (0)	1 (3)
Used with verbal autopsy	14 (17)	0 (0)	12 (38)
Maximum percent of deaths classified as "other" using this system			
< 20 %	39 (48)	19 (53)	17 (53)
≥ 20 %	10 (12)	3 (8)	6 (19)
No "other" category	27 (33)	12 (33)	9 (28)
“Other” category but no data available	5 (6)	2 (6)	0 (0)
Maximum percent of deaths classified as "unexplained" using this system			
< 20 %	25 (31)	12 (33)	13 (41)
≥ 20 %	38 (47)	18 (50)	14 (44)
No "unexplained" category	11 (14)	4 (11)	4 (13)
“Unexplained” category but no data available	7 (9)	2 (6)	1 (3)
Allows the type of data available for assigning COD to be recorded	7 (9)	7 (19)	0 (0)
Allows recording the level of certainty of the data	34 (42)	19 (53)	12 (38)
For systems including SB	55	29	18
Requires recording whether the stillbirth was antenatal vs intrapartum			
Yes	16 (29)	8 (28)	5 (28)
No	14 (26)	8 (28)	5 (28)
Partially	25 (46)	13 (45)	8 (44)
For systems including both SB and NND	40	20	14
Includes guidelines that require distinguishing between SB and NND			
Yes	13 (33)	6 (30)	5 (36)
No	22 (55)	12 (60)	6 (43)
Unclear	5 (13)	2 (10)	3 (21)
Has separate categories for SB and NND			
Yes, all	9 (23)	2 (10)	6 (43)
No	11 (28)	7 (35)	2 (14)
Some	20 (50)	11 (55)	6 (43)
For systems allowing associated factors to be recorded	23	13	6
Distinguishes associated factors from causes of death			
Yes	11 (48)	6 (46)	6 (67)
No	10 (44)	7 (54)	1 (17)
Unclear	9 (9)	0 (0)	1 (17)

NOTE: Column percentages used. Due to rounding, totals may be slightly different from 100 %

#### Reasons for system creation

Authors of 27 of the 73 systems which we were able to identify as either new or modified provided no rationale for the creation or modification of the systems. Reasons provided for the remainder focused on adding features [25] and missing categories [26, 27], accommodating new knowledge on causation and increasing accuracy [28], reaching new audiences (e.g. in low-and middle-income countries, LMIC) [29], addressing underlying causes [5, 8, 11, 30, 31], providing rules and/or definitions [7, 8, 26, 29, 32–35], or reducing the proportion of “unexplained” deaths [27, 32, 35–38]. Some found the inclusion of both SB and NND to be a shortcoming to be addressed (through creation of SB-only or NND-only systems) [33], while others felt that limiting systems to SB only or NND only was a shortcoming to be addressed (through creation of a system for both SB and NND) [8, 35]. There was a similar difference of opinion regarding whether hierarchy was a shortcoming to be addressed through creation of a non-hierarchical system [39], or a useful feature to incorporate into a new system [29].

### Overview of system characteristics

Characteristics of the 81 included systems are presented in Table 1. The characteristics that were most common among the systems regardless of whether used in high-income countries (HIC) only or LMIC only were: (i) exclusion of fetal growth restriction (FGR), intrauterine growth restriction (IUGR) and small-for-gestational age (SGA) from the list of causes (75 % and 88 % of HIC-only and LMIC-only systems, respectively); (ii) requiring a single cause of death to be recorded (81 % and 72 %); (iii) ten or fewer causes at the top level (72 % and 88 %); (iv) not requiring recording of the type of data used to assign causes (81 % and 100 %); (v) not using ICD codes (92 % and 75 %); (vi) not having been tested for reliability (86 % and 88 %); (vi) use in just one country (83 % and 94 %); (vii) unavailable in e-format (94 % and 97 %); and (viii) unavailable in multiple languages (97 % and 100 %).

In addition to these, the characteristics that were most common among the 36 systems used only in HIC were: (i) non-hierarchical; and (ii) not having been used with verbal autopsy. Characteristics most common among the 32 systems used only in LMIC included: (i) lack of rules for assigning causes of death; (ii) lack of guidance on how to access data from systems; (iii) no inclusion of associated factors; and (iv) used to classify fewer than 500 deaths (among publications included in our search 2009–2014).

### Comprehensiveness of systems

#### Types of deaths included

Systems classifying both SB and NND were most common, with just under half the systems classifying both types of death. Next most common were systems classifying just NND (around one-third of systems) (see Table 1). There was a difference in type of death classified according to region of use. Of the 36 systems used in HIC only, over half classified both types of death, and one quarter classified SB only. SB-only systems were less common among the 32 systems used in LMIC only: 14 systems classified both SB and NND death and 14 classified NND only, while just four classified SB only.

Of the 55 systems that included SB, a minority (n = 16, 29 %) required distinguishing between antepartum (AP) and intrapartum (IP) SB, with similar results across HIC and LMIC settings. For the 40 systems including both SB and NND, more than half (n = 22) provided no guidelines or rules for distinguishing between SB and NND, and 11 had no categories that were clearly either SB or NND (see Table 1).

#### Associated factors

Twenty-three systems (28 %) allowed associated factors to be recorded (see Table 1). This feature was more common among HIC-only systems (13 of the 36 systems) than LMIC-only systems (six of the 32 systems). Less than half (n = 11) of systems allowing associated factors clearly distinguished them from causes of death.

### Extent of use of all systems

#### Regions of origin and use

**Fig. 4:**
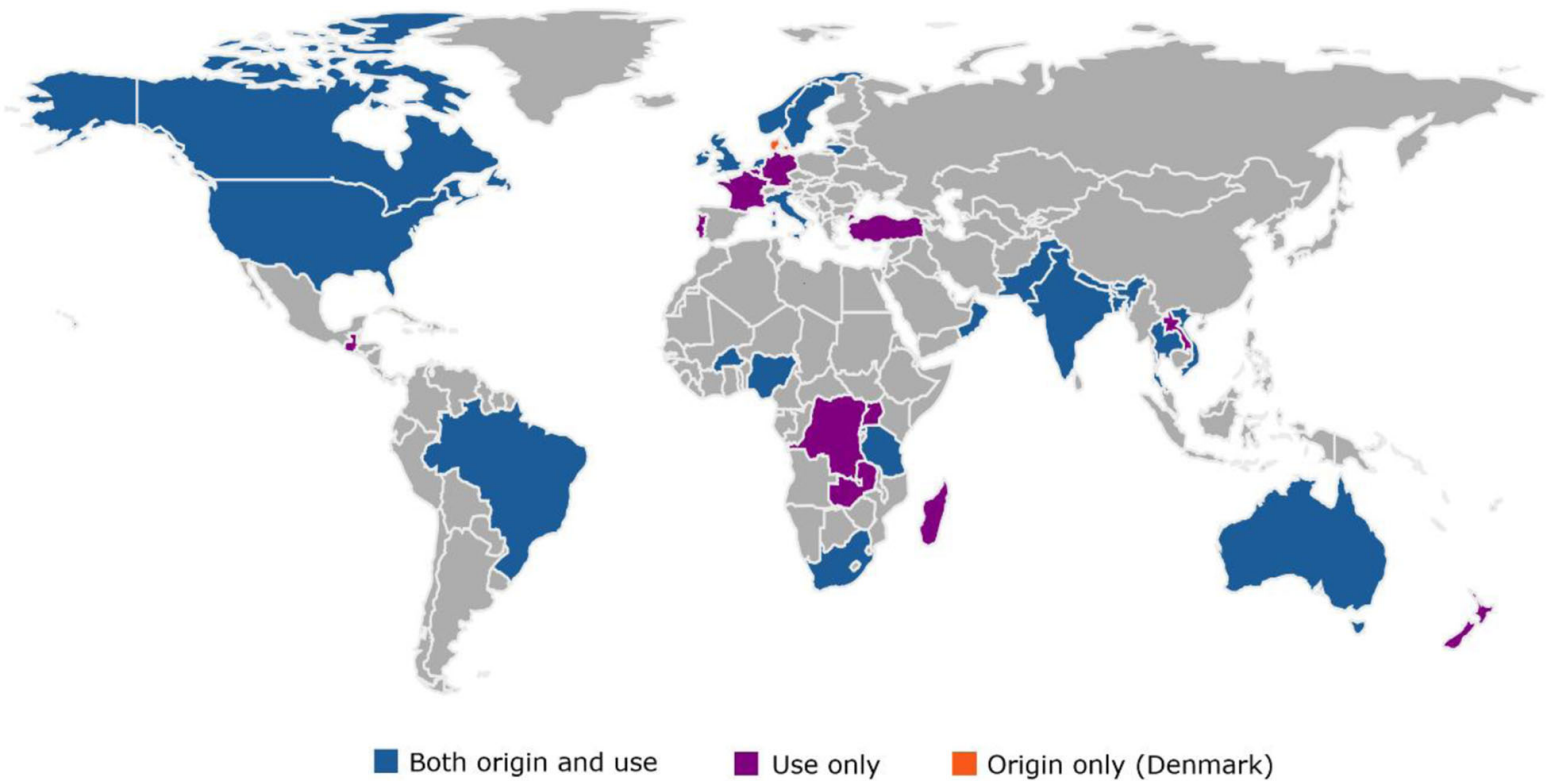
Classification systems for causes of stillbirths and neonatal deaths, 2009–2014: Countries of origin and use

Systems were created or modified in 28 countries on six continents, the majority (65 %) in HIC, and were used in a total of 40 countries (see Fig. 4). Of the 53 systems created in HIC, most (68 %) were used only in HIC. Of the 28 systems created in LMIC, the majority (86 %) were used only in LMIC. Half of the 81 systems were used only in the publications which presented them. Most systems (74 %) were used in just one country, and five systems were described but not used. Four systems were used to report global data; other than these, the largest number of countries in which any system was used was seven (by Wigglesworth 1980 and Gardosi 2005-ReCoDe) (see Additional file 7). About one-fifth of the 81 systems (n = 17) were national, including 12 systems used in eight HIC and five systems used in five countries in Asia, Africa, and South America (see Additional file 8).

#### Systems used in highest-burden settings

Included systems were used in only about half of the highest-burden countries (six of the top 11 highest-NND burden countries and six of the top 10 highest-SB burden countries) (see Additional file 9). This included just one national system, used in Bangladesh. Specifically, no systems were found to be used in the two highest-burden countries, China and India (though the ICD has been used to classify perinatal deaths in China [40]). Other than systems used to estimate global causes, only two systems were used in more than one highest-burden country: Engmann 2012 [39] (in Pakistan and the Democratic Republic of the Congo, DRC) and Wigglesworth 1980 [7] (in Pakistan and Bangladesh).

#### Number of deaths classified

According to published reports of system use, 49 of 81 systems (60 %) had been used to classify fewer than 500 deaths, including 17 of the 36 systems used only in HIC (47 %) and 26 of the 32 systems used only in LMIC (81 %; see Table 1). Just under one third of systems (28 %) were used to classify 1000 or more deaths: 12 of the 36 systems used only in HIC (33 %) and just four of the 32 systems used only in LMIC (13 %) (see Table 1).

Other than global systems and systems that were not used, systems classified between 14 and 47,238 deaths. The total deaths classified by systems (excluding global systems) between 2009 and 2014 was just under 234,000, representing less than 1 % of all SB and NND globally in this period (assuming 2.6 million stillbirths and 2.7 million neonatal deaths annually [1, 2]) (see Table 2 for data on numbers of deaths classified by widely used systems; other data not shown).

### Most widely used systems and their selected characteristics

Systems used in more than one country and/or to classify 1000 or more deaths were considered to be “widely used” (see Additional file 4 for the results of sensitivity analysis of these cut-offs). It is worth noting that national systems in countries with small numbers of perinatal deaths, such as Bhutan and Wales, were thus not considered to be widely used, though they may cover a high percentage of deaths within their context. By this definition, 27 systems (33 %) were widely used, including almost half of the 17 national systems (see Table 2). Thirteen of the 27 most widely used systems classified both SB and NND, 10 classified NND only and four classified SB only. Most (about 70 %) of the widely used systems were not hierarchical. Nearly one-third of the 17 widely used systems which included SB did not distinguish at all between AP and IP SB.

**Table 2:** Widely used classification systems for causes of stillbirth and neonatal death, 2009–2014: Selected characteristics

	Country of origin^a^	Region and countries of use (2009–2014)^b^	# deaths classified (2009–2014)^b^	Hier	IP vs AP	SB vs NND cats	Single cause	# causes	# levels	Ass'd factors	Ass'd factors vs causes	Defs	Rules	Max % unex
Systems classifying both SB and NND														
CMACE 2010-maternal & fetal [36]	UK	HIC (UK)	6,804	No	Yes	No	No	13	2	Yes	No	Some	Yes	39 %
CMACE 2011-maternal & fetal [46]	UK	HIC (UK, Wales)	9,786	No	Yes	No	Yes	12	3	Yes	No	No	No	51 %
Cole 1986 [26]	UK	Both (Nigeria, Netherlands)	345	Partly	No	No	Yes	10	2	No	n/a	Yes	Yes	55 %
Engmann 2012 [39]	USA	LMIC (Guatemala, DRC, Zambia, Pakistan)	252	No	Partial	Yes, all	Yes	7.5^c^	1	No	n/a	No	No	12 %
Flenady 2009-PSANZ-PDC [28]	Australia	Both (Australia, Vietnam, New Zealand, Madagascar)	13,416	Partly	Partial	No	Yes	7	4	Yes	Yes	Some	Yes	54 %
Frøen 2009-Codac [11]	Norway	HIC (Norway, Italy, Wales)	872	Partly	Yes	Some	Yes	10	3	Yes	Yes	Some	Yes	53 %
Korteweg 2006-Tulip [35]	Neth.	HIC (Neth.)	3,603	No	No	No	Yes	6	3	Yes	Yes	Some	Yes	23 %
Manandhar 2010 [47]	Nepal	LMIC (Nepal)	1,272	Unclear	Yes	Yes, all	Yes	9^c^	1	No	n/a	Yes	No	10 %
National Services Scotland 2013-FIGO [27]	Scotland	HIC (Scotland)	1,249	No	No	Yes, all	Yes	4	1	No	n/a	No	No	100%^d^
MRC 2002-PPIP [48]	South Africa	LMIC (South Africa)	47,238	No	Partial	Some	Unclear	9.5^c^	1	Yes	Yes	Some	No	35 %
Wigglesworth 1980 [7]	UK	Both (Turkey, Bangladesh, UK, Ireland, Nepal, Pakistan, Brazil)	4,558	No	Partial	Some	Yes	5	1	No	n/a	Some	Yes	56 %
Winbo 1998-NICE [31]	Sweden	LMIC (Tanzania)	2,494	Yes	No	Some	Yes	13	1	No	n/a	Yes	No	46 %
Wood 2012 [49]	UK	HIC (Scotland)	8,332	No	Yes	Some	Yes	2	2	No	n/a	Yes	No	60 %
Systems classifying SB only														
Dudley 2010-INCODE [34]	USA	HIC (Canada, USA)	1,075	No	Partial	n/a	No	7	4	No	n/a	Some	Yes	n/a^e^
Gardosi 2005-ReCoDe [37]	UK	Both (Italy, UK, France, Portugal, New Zealand, Germany, Brazil)	25,779	Yes	Partial	n/a	No	9	2	Yes	No	Some	Yes	26 %
Seaton 2012 [50]	UK	HIC (UK)	21,352	No	Partial	n/a	Yes	9	1	No	n/a	Yes	No	41 %
Varli 2008-Stockholm [33]	Sweden	HIC (Sweden)	1,089	No	No	n/a	Yes	17	2	Yes	No	Yes	Yes	19 %
Systems classifying NND only														
Black 2010-CHERG [51]	USA	Global	>1 million	No	n/a	n/a	Yes	8	1	No	n/a	Some	Yes	23 %
CMACE 2010-neonatal [36]	UK	HIC (UK, Ireland)	7,717	No	n/a	n/a	No	10	2	Yes	No	Some	Yes	6 %
Cole 1989-ICE [52]	UK	HIC (Canada, USA)	38,692	No	n/a	n/a	Yes	8	1	No	n/a	Yes	Yes	15 %
Flenady 2009-PSANZ-NDC [28]	Australia	HIC (Australia, New Zealand)	3,449	No	n/a	n/a	Yes	11	3	Yes	Yes	Some	Yes	n/a^f^
Lawn 2006-CHERG [53]	South Africa	Both (sub-Saharan Africa, Laos, Uganda); also global	>1 million	Yes	n/a	n/a	Yes	7	1	No	n/a	Yes	Yes	23 %
Lawn 2012 [54]	South Africa	Global	>1 million	No	n/a	n/a	Yes	5	1	No	n/a	No	No	n/a^f^
Lawn 2010 [55]	South Africa	Global	>1 million	No	n/a	n/a	Yes	5	1	No	n/a	No	No	n/a^f^
Rocha 2011 [56]	Brazil	LMIC (Brazil)	2,893	No	n/a	n/a	Unclear	6	2	No	n/a	No	No	20 %
Smith 2010 [57]	UK	HIC (UK)	18,524	Yes	n/a	n/a	Yes	10	1	No	n/a	No	No	5 %
Winter 2013-Rwanda [58]	Rwanda	LMIC (Bhutan, Rwanda)	628	No	n/a	n/a	Yes	7	1	No	n/a	No	No	n/a^f^

*Hier* Hierarchical or not, *IP vs AP* Requires distinguishing antepartum from intrapartum stillbirth, *SB vs NND cats* Includes separate categories for stillbirths and neonatal deaths, *Single cause* Requires single cause to be identified, # *causes* Number of causes at top level, *Ass*’*d factors* Allows associated factors to be recorded, *Ass*’*d factors vs causes* Requires associated factors and causes to be distinguished from one another, *Defs* Includes definitions for all causes, *Rules* Includes guidelines for assigning cause of death, *Max* % *unex* Maximum percent of deaths classified as “unexplained” (see Additional file 10 for more detail)

NOTE: All data other than region/countries of use and number of deaths classified was taken from reference papers for included systems, which are cited in the first column. “Widely used” is defined as used to classify >1000 deaths and/or in 2+ countries between 2009 and 2014

^a^Defined as country of first affiliation of first author of reference paper

^b^Region and countries of use and numbers of deaths classified all taken exclusively from included papers between 2009 and 2014 that reported use of the included systems

^c^Average taken when there was more than one set of levels (e.g. one for stillbirths and one for neonatal deaths)

^d^The system only allocates stillbirths to one of two “causes”, both of which are considered to be “unexplained”; see Additional file 10 for more detail

^e^ The system has a category for “unexplained” but there was no data reported

^f^ These systems have no category for “unexplained”

The majority of the widely used systems (78 %) required identifying a single cause of death. Ten allowed associated factors to be recorded, although this varied depending on which types of deaths were classified, with two of the four widely used SB-only systems and two of the 10 widely used NND-only systems allowing associated factors. Most of the 27 widely used systems (70 %) provided definitions for at least some causes of death, though only eight systems provided definitions for all causes. About half gave some description of how cause of death should be assigned (see Table 2).

Widely used systems differed from less used systems in several respects. They were more likely to: (i) be used in both HIC and LMIC (eight of 27 systems, or 30 %, as opposed to none of the 54 less used systems); (ii) have been tested for reliability (22 % vs 7 % respectively); (iii) be available in e-format (11 % vs none); (iv) record the degree of certainty of the cause of death assigned (48 % vs 39 %); (v) record the type of data available for assigning cause of death (19 % vs 4 %); (vi) provide definitions for some or all causes of death (70 % vs 50 %); (vii) provide rules for assigning cause of death (52 % vs 35 %); and (viii) allow associated factors (37 % vs 24 %). Widely used systems that included both SB and NND were also more likely to clearly distinguish the two types of death (six of the 13 widely used systems including both SB and NND vs seven of the 27 less used systems including both types of deaths).

Widely used systems were less likely to: (i) be used in LMIC only (22 % of widely used systems versus 48 % of less used systems); and (ii) have recorded a maximum proportion of deaths classified as “unexplained” that was less than 20 % (22 % vs 35 %) (data not shown).

### Accessibility and relevance

The majority of systems (n = 66, 82 %) provided no guidance on how potential users might access data from their systems. Three systems were available in e-format (as defined by availability of a form that could be filled in online). Just one system was available in more than one language (English and Lithuanian). Fourteen systems (17 %) had been used with verbal autopsy (see Table 1).

### Identification of underlying causes

#### Number of causes and levels

Systems had from one to four levels (see Fig. 1 for definition of this term), with a mean of 1.8 levels. Just over half had more than one level. Nine of the 36 HIC-only systems (25 %) versus three of the 32 LMIC-only systems (10 %) had three or more levels. The range of number of causes at the top level was two to 40, with a median of 8.2 causes. Most systems (n = 67, 83 %) had 10 or fewer causes at the top level. Of the 14 systems with more than 10 causes at the top level, 10 were used only in HIC. Most systems (n = 64, 79 %) required that a single cause of death be recorded, with similar results for HIC-only and LMIC-only systems (see Table 1).

#### Hierarchy

Most systems (n = 53, 65 %) were not hierarchical, while just under one-quarter were completely hierarchical. Hierarchy was more common among the 32 systems used only in LMIC (just under one-third of these were completely hierarchical) than among the 36 systems used only in HIC (14 % were completely hierarchical) (see Fig. 1 for definition of terms and Table 1 for data).

#### Percent “other” and “unexplained”

Around two-thirds of systems (n = 54) had at least one category for grouping causes not defined elsewhere in the system as “other” (see Table 1). For most of these systems (72 %), the maximum proportion of deaths classified as “other” was less than 20 %, a finding that was similar for both HIC-only and LMIC-only systems. The range of the maximum proportion of deaths classified as “other” was 0 % [41] to 68 % [47], with an average of 14 % and a median of 8 % (for systems with at least one “other” category and available data). The range of proportion of deaths classified as “other” was somewhat narrower for SB-only (1–48 %) and NND-only systems (0–54 %) than for systems including both types of deaths (1–68 %) (see Additional file 10).

The majority of systems (n = 70, 86 %) also had categories for “unexplained” deaths. Of these 70 systems, just 36 % had a maximum proportion of deaths classified as “unexplained” that was less than 20 %. Slightly more LMIC-only systems than HIC-only had this relatively low proportion of deaths classified as “unexplained" (46 % of LMIC-only versus 38 % for HIC-only systems, including only systems with at least one “unexplained” category). The range was 0 % [42] to 100 % (the FIGO system as used in [27]),^2^ with an average of 29 % and a median of 23 %. (The mean and median were virtually unchanged when the outlier of 100 % was excluded.) The range of proportion of deaths classified as “unexplained” was narrowest for NND-only systems (0–30 %) and widest for systems including both types of deaths (6–100 %; excluding the slight outlier of 100 %, the range was 0–81 %). See Additional file 10 for details and a list of terms that were included in the assessment of the proportion of deaths classified as “other” and “unexplained”.

### Reliability

#### Reliability testing

Only 10 systems (12 %) were tested for reliability between 2009 and 2014 (see Table 1), about half of these only internally (by the teams which had developed the systems). Eight of the 10 tested systems originated in HIC. Three groups tested systems other than their own, and four systems were tested more than once. The overall Kappa ranged from .35 (poor agreement) (for Cole 1986 [26]) to .93 (excellent agreement) (for Korteweg 2006-Tulip [35]); all but one of the Kappa values were over .50 (fair to excellent) (see Additional file 11). The range for external Kappas (Kappa values from testing by teams which had not developed the systems being tested) was .35–.93 and the range for internal Kappas (Kappa values from testing by teams which had developed the systems being tested) was .51–.89. The 59 modified systems were much less likely to have been tested for reliability than the 14 new systems (9 % v 36 %, respectively).

#### Availability of definitions and rules

Just 23 of the 81 systems (28 %) provided definitions for all causes of death, and 33 (41 %) provided some description of how to assign causes of death (see Table 1). Sixteen of the 32 systems used only in LMIC (50 %), and 14 of the 36 systems used only in HIC (39 %), provided no definitions for causes. The majority of LMIC-only systems (n = 23, 72 %) and HIC-only systems (n = 20, 56 %) provided no guidance on assigning cause of death. Only seven of 81 systems (9 %) allowed recording of the type of data used to assign cause of death, all of them HIC-only systems.

### System alignment with the ICD

Seventeen of the included systems (21 %) used ICD codes; this was more common among LMIC-only systems (25 %) than HIC-only systems (8 %) (see Table 1).

## Discussion

We reviewed contemporary classification systems used for causes of stillbirths and neonatal deaths globally, to inform development of the new ICD-PM. We found a large number of systems in addition to the ICD, with widely varying characteristics and limited reach in terms of numbers of deaths classified, especially in highest-burden countries.

The most comprehensive review of classification systems prior to this one, by Gordijn et al., described 35 systems published in English developed between 1954 and 2006 [8]. In 2009, Flenady et al. identified and tested six contemporary systems commonly used for stillbirth in HIC using independent teams across a number of countries [20]; a publication by Frøen et al. on challenges of data collection reviewed 11 systems [19]. In 2014, a systematic review of studies reporting factors associated with stillbirth in LMIC found just seven systems used [21]. We identified far more systems developed and used than these previous reviews. While our comprehensiveness (including no language restriction) may partially explain this difference, the inclusion of “modifications”, even if minor, is likely the major reason. We did this both because even slight modification may affect data comparability, and because modification may reflect users’ perceptions of the inadequacy of available systems. We also included systems for both stillbirth and neonatal death, whereas most previous reviews focused on stillbirth.

While the overarching aim of all perinatal death classification systems is to understand causes to enable prevention, systems had multiple specific purposes and rationales, including national tracking (e.g., MRC 2002-PPIP [50]), in-depth investigation (e.g., Flenady 2009-PSANZ-PDC [28]), research (e.g., Dudley 2010-INCODE [34]), or more generally to overcome shortcomings of existing systems and meet context-specific needs [4, 31, 33] (see Additional file 12). Numerous incompatible systems reduces the utility of the data of each [43], yet few papers describing new or modified systems mentioned other systems. Only one-third of systems were “widely used” by our definition (see Table 2), and systems collectively classified only a small proportion of perinatal deaths globally between 2009 and 2014 (other than those estimating global causes, e.g. CHERG for NND only); none were classified in six of the 12 highest-burden (LMIC) countries. National systems were used in only a few countries (see Additional file 8), and there were none in the two highest-burden HIC (the US and Russia). Low coverage may be due to lack of the required data or poor system accessibility, both of which may reflect systems’ unsuitability, especially for low-resource settings. The size of the burden itself, requiring allocation of scarce resources to healthcare, may place a high opportunity cost on the resources required for classification, even in high-resource settings. Coverage may also be hampered by a silo effect, with over half of systems only used by the teams that created or modified them, and most only used in the regions where they were created, possibly because many systems are context-specific. For instance, there are more NND-only systems in LMIC, a situation which may be driven by the relative lack of SB data and attention to SB in LMIC. With nearly twice as many systems created in HIC as in LMIC, this suggests potential LMIC users may also have less choice in terms of available, locally relevant systems. In particular, limited diagnostic capacity in low-resource settings may make some systems based on pathology findings impossible to use.

The multiple systems reflect many challenges for the uptake of a system aimed at global application. This review suggests ways to increase global uptake. Characteristics found to be common among all systems (e.g. requiring a single cause of death and lacking hierarchy), and among the most widely-used systems (e.g. availability of rules and definitions), could be considered proxies for what users expect in an effective system. The characteristics that were rarest (e.g. using ICD codes and having been tested for reliability) may reflect not only user preferences, but also the resources available to users. A globally acceptable system might also benefit from incorporating the most common characteristics of systems used only in LMIC (to increase uptake across settings), and from exploring in greater depth than was possible in this study the reasons why certain features (e.g. reliability testing) were quite uncommon. A global system must accommodate not only low levels of data in poorer settings but also more detailed data in HIC settings, or other regions with access to better diagnostics [44]. Disseminating a system widely, removing language barriers, offering electronic as well as paper-based data collection, training users, assessing system reliability, and addressing users’ concerns with established systems would increase acceptance and uptake of any system intended for global use, including by governments. Systems’ broad albeit thin reach also presents opportunities; for instance, a new global system could be introduced through existing channels for classification.

The ICD is the global standard for assigning diagnoses. It is used for reporting deaths in 117 countries, sometimes including perinatal deaths, for example in three of the highest burden countries—China, Tanzania and Bangladesh [32, 40, 45]. However, perinatal deaths, in particular stillbirths, remain poorly captured and classified; this is a driving factor in the WHO’s work to create the ICD-PM. Many systems are incompatible with the ICD’s key principles, such as identification of a single cause of death, use of ICD codes, incorporation of associated factors, and distinguishing between IP and AP, and between SB and NND. This may be in part due to low awareness of its importance, but is more likely to be due to the ICD’s limited utility for classification of stillbirths. It is hoped that future revisions of the ICD will address this limitation. A particular concern is the low percentage of systems that require recording the timing of deaths (IP vs AP). This information is among the most basic and is obtainable even in low-resource settings, yet was only required by 16 of the 55 systems that include SB, reflecting the larger issue of insufficient data on IP stillbirths worldwide, despite the huge burden and preventability of most of these deaths [2].

This review had some limitations. The comprehensive search notwithstanding, some systems may not have been identified; no regional databases were searched. This would have led to an underestimate of the true number of systems, possibly weighted toward those in LMIC. The quality of included publications was not assessed, so data used to assign values for percent of deaths classified as “other” and “unexplained” and number of deaths classified was likely of varying quality. For national systems, since only the most recent publication within 2009–2014 was included, the number of deaths classified may be an underestimate. However, this would likely not have affected our findings significantly. Data for some variables were difficult to ascertain, for instance the number of languages in which a system was available, possibly leading to non-differential misclassification of systems for some variables. We were unable to review findings with system authors or double-extract data from non-English publications (6 % of included publications).

## Conclusions

Stillbirth and neonatal death deprive millions of babies of their right to grow and develop, bereaving their parents and other family members and affecting millions of caregivers. Though this burden is decreasing, progress is slow. Greater effort must be made, through increased attention from policy-makers, bolder partnerships across the reproductive, maternal, and child health spectrum, country leadership, and innovative programs to scale up effective interventions. Classification of causes is critical to this effort. Whether directly or indirectly, the ultimate aim of classification is to provide data that can be useful in reducing stillbirth and neonatal death. A prime example of how classification systems can be useful is in the recording of stillbirth timing—whether antepartum or intrapartum. This data should be generally available even in low-resource settings and is actionable, even amidst the chaos of multiple systems.

This systematic review provides a comprehensive summary of the landscape of contemporary classification systems for stillbirths and neonatal deaths to inform the development of a globally acceptable approach for the accurate determination of causes of death. In part two of the study, we assess the alignment of the 81 identified systems with expert-identified characteristics for a globally acceptable classification system [23]. We hope that this study will ultimately prove useful not only to researchers and practitioners, but also to bereaved families in all countries who want to know “what happened”.

## Additional files

Additional file 1: PRISMA checklist. (DOCX 51 kb)
Additional file 2: Decision tree for inclusion/exclusion. (DOCX 45 kb)
Additional file 3: List of variables extracted. (DOCX 46 kb)
Additional file 4: Sensitivity of number of “widely used” systems to cut-offs for number of countries in which used and number of deaths classified. (DOCX 46 kb)
Additional file 5: All included publications with reason for inclusion and author intent. (DOCX 223 kb)
Additional file 6: Year of creation/modification of classification systems for causes of stillbirth and neonatal death developed or used between 2009 and 2014. (DOCX 48 kb)
Additional file 7: Countries in which more than one system was used to classify causes of stillbirths and/or neonatal deaths, 2009-2014. (DOCX 45 kb)
Additional file 8: National classification systems for causes of stillbirth and neonatal death in use 2009-2014. (DOCX 53 kb)
Additional file 9: Classification systems for causes of stillbirth and neonatal death used in highest-burden countries, 2009-2014. (DOCX 83 kb)
Additional file 10: Maximum percent of deaths classified as “other” and “unexplained” by classification systems according to type of death classified. (DOCX 62 kb)
Additional file 11: Kappa scores from reliability testing of classification systems for causes of stillbirth and neonatal death in use 2009-2014. (DOCX 66 kb)
Additional file 12: Selected shortcomings of existing systems and rationale for development of new systems/modification of existing systems. (DOCX 44 kb)

## Abbreviations

AP: Antepartum
CHERG: Child Health Epidemiology Reference Group
CMACE: Centre for Maternal and Child Enquiries
COD: Cause of death
Codac: Causes of death and associated conditions
DHS: Demographic and Health Surveys
DRC: Democratic Republic of the Congo
FGR: Fetal growth restriction
FIGO: International Federation of Gynaecology and Obstetrics
HIC: High-income countries
ICD: International Classification of Diseases
ICD-PM: International Classification of Diseases for Perinatal Mortality
ICE: International collaborative effort
INCODE: Initial Causes of Fetal Death
IP: Intrapartum
IUGR: Intrauterine growth restriction
LMIC: Low- and middle-income countries
MAIN: The Maternal, Antenatal, Intrapartum & Neonatal Classification System for Perinatal Deaths
MRC: Medical Research Council
NICE: Neonatal and Intrauterine Death Classification according to Etiology
NIPORT: National Institute of Population Research and Training
NND: Neonatal death
PMMRC: Perinatal and Maternal Mortality Review Committee
PPIP: Perinatal Problem Identification Programme
PSANZ-NDC: Perinatal Society of Australia and New Zealand Neonatal Death Classification
PSANZ-PDC: Perinatal Society of Australia and New Zealand Perinatal Death Classification
ReCoDe: Relevant condition at death
SB: Stillbirth
SGA: Small for gestational age
WHO: World Health Organization
WiSSP: Wisconsin Stillbirth Service Program

## Footnotes

1 There was not a one-to-one correspondence between included publications and included systems (many publications included more than one system; multiple publications used the same system); hence search results do not demonstrate the total number of systems found.

2 The system was National Services Scotland 2013-FIGO, [27] which only allocates stillbirths to one of two “causes”, SB weighing 1000 g + and normally formed SB weighing 500 g+, both of which were included as “unexplained” causes in the BMC Supplement companion paper that we used as our guide (Reinebrant H, Zheyi T, Wojcieszek AM, Coory M, Gardener G, Lourie R et al. Causes of stillbirth globally – burden in high- and low-resource settings: in preparation).
