# Supplementary material for: Seeking order amidst chaos: a systematic review of classification systems for causes of stillbirth and neonatal death, 2009–2014
Source: BMC Pregnancy Childbirth. 2016 Oct 5;16:295. doi: 10.1186/s12884-016-1071-0 (PMC5053068; doi:10.1186/s12884-016-1071-0)
Supplement: Additional file 2: — Decision tree for inclusion/exclusion. (DOCX 45 kb) [file 12884_2016_1071_MOESM2_ESM.docx]

## Additional file 2

### Decision tree for inclusion/exclusion

Title/abstract indicates new/modified system for stillbirth (SB)/neonatal death (NND)/perinatal death classification is described?

- IF yes, INCLUDE
- IF no OR unsure:
  - Title/abstract indicates data on all causes of death (COD) of a group of >3 cases of SB/NND/perinatal death are included (whether or not a classification system is used)?
- IF yes, INCLUDE
- IF no: EXCLUDE
- IF unsure: expert to decide.

Excluded:

- post-neonatal deaths
- COD for specific COD-groups, e.g. sudden infant death syndrome (SIDS)
- COD data given as rates only

Included:

- COD for groups not defined by a single COD, e.g. deaths in infants with Down Syndrome, nighttime deaths
