# Supplementary material for: Seeking order amidst chaos: a systematic review of classification systems for causes of stillbirth and neonatal death, 2009–2014
Source: BMC Pregnancy Childbirth. 2016 Oct 5;16:295. doi: 10.1186/s12884-016-1071-0 (PMC5053068; doi:10.1186/s12884-016-1071-0)
Supplement: Additional file 3: — List of variables extracted. (DOCX 46 kb) [file 12884_2016_1071_MOESM3_ESM.docx]

## Additional file 3

### List of variables extracted

#### Variables used to describe basic system characteristics:

1. First author of reference paper
2. Country of first affiliation of first author
3. Year of publication of reference paper
4. Name of system if any
5. Whether modified or new
6. Type of modification (minor or major) if relevant
7. System from which modified if relevant
8. Reference for original system if relevant
9. Author’s intent regarding whether modified, new, or use of existing system
10. Purpose of system creation (text from reference paper)
11. Whether there is a category for “other”
12. Maximum % “other” recorded
13. Whether there is a category for “unexplained”
14. Maximum % “unexplained” recorded
15. Citations for these uses, if any
16. References for same, if relevant
17. Whether certainty of the data is recorded
18. Whether tested for ease of use
19. Results, if so
20. References for same, if relevant
21. Citation for reference paper

#### Variables to assist in assessing alignment with expert-identified characteristics for a globally acceptable system:

*Comprehensiveness:*

1. SB-only, NND-only or combined
2. Whether rules for distinguishing between SB and NND are provided
3. Whether all or some categories are clearly either SB or NND
4. Whether associated factors are recorded
5. Whether associated factors are distinguished from causes of death
6. Whether AP and IP are fully or partially distinguished

*Extent of use:*

1. Region (high-income country, HIC, or low-income country, LMIC) of country of affiliation of first author
2. Countries in which used
3. Regions of use (HIC, LMIC or both)
4. National system or not
5. Number of deaths classified

*Accessibility:*

1. Whether access to data available
2. Whether available in e-format
3. Languages in which available
4. Whether used with verbal autopsy

*Identification of underlying causes:*

1. How many causes are in the top level
2. How many levels
3. Whether fully or partially hierarchical
4. Whether a single cause must be recorded
5. Whether causes include fetal growth restriction (FGR), intrauterine growth restriction (IUGR) and/or small for gestational age (SGA)

*Reliability:*

1. Whether tested for reliability
2. Results, if so
3. References for same, if relevant
4. Whether definitions for some or all causes provided
5. Whether rules provided
6. Whether type of data used to assign COD is recorded

#### Variable to assess International Classification of Diseases (ICD) alignment:

1. ICD codes used?
