# Supplementary material for: Seeking order amidst chaos: a systematic review of classification systems for causes of stillbirth and neonatal death, 2009–2014
Source: BMC Pregnancy Childbirth. 2016 Oct 5;16:295. doi: 10.1186/s12884-016-1071-0 (PMC5053068; doi:10.1186/s12884-016-1071-0)
Supplement: Additional file 4: — Sensitivity of number of “widely used” systems to cut-offs for number of countries in which used and number of deaths classified. (DOCX 46 kb) [file 12884_2016_1071_MOESM4_ESM.docx]

## Additional file 4

### Sensitivity of number of “widely used” systems to cut-offs for number of countries in which used and number of deaths classified

| Variable | Range | “Widely used” if | Number of widely used systems |
| --- | --- | --- | --- |
|  |  | Used in 2+ countries and/or on >1000 deaths (original cut-off) | 27 |
| # countries in which used | (0, 7) (other than global) | Used in 1+ countries and/or on >1000 deaths | 76 |
|  |  | Used in 3+ countries and/or on >1000 deaths | 25 |
| # deaths classified | (0, >1 million) | Used on >500 deaths and/or in 2+ countries | 34 |
|  |  | Used on >1500 deaths and/or in 2+ countries | 24 |

Methodology: The number of widely used systems was calculated for:

- Our definition of “widely used” (used in two or more countries or on more than 1000 deaths)
- Holding the “number of deaths” variable constant, but varying the number of countries in which used: for systems used in at least one country, and for systems used in at least three countries
- Holding the “number of countries in which used” variable constant, but varying the number of deaths for which used: for systems that were used to classify over 500 deaths, and for systems that were used to classify over 1500 deaths
