# Supplementary material for: Seeking order amidst chaos: a systematic review of classification systems for causes of stillbirth and neonatal death, 2009–2014
Source: BMC Pregnancy Childbirth. 2016 Oct 5;16:295. doi: 10.1186/s12884-016-1071-0 (PMC5053068; doi:10.1186/s12884-016-1071-0)
Supplement: Additional file 5: — All included publications with reason for inclusion and author intent. (DOCX 223 kb) [file 12884_2016_1071_MOESM5_ESM.docx]

## Additional file 5

### All included publications with reason for inclusion and author intent

| **First author, year** | **Reason for inclusion** | **Author intent** | **Name(s) of new or modified system(s), if any (name of country, if national)** | **System(s) used, if any** |
| --- | --- | --- | --- | --- |
| Abdellatif 2013 [[1](#_ENREF_1)] | M | U |  |  |
| Abha 2011 [[2](#_ENREF_2)] | M | U |  |  |
| Aggarwal 2011 [[3](#_ENREF_3)] | M, T | M |  |  |
| Aggarwal 2013 [[4](#_ENREF_4)] | N, T | NSI^f^ |  |  |
| Akkhavong 2009 [[5](#_ENREF_5)] | U |  |  | Lawn 2006-CHERG |
| Aminu 2014 [[6](#_ENREF_6)] | U |  |  | Gardosi 2005-ReCoDe |
| Amouzou 2010 [[7](#_ENREF_7)] | U |  |  | NIPORT 2005-Bangladesh |
| Andriamandimbison 2013 [[8](#_ENREF_8)] | U |  |  | Flenady 2009-PSANZ-PDC |
| Bakketeig 2011 [[9](#_ENREF_9)] | U |  |  | Hinderaker 2003 |
| Basys 2014 [[10](#_ENREF_10)] | M | U | (Lithuania national) |  |
| Black 2010 [[11](#_ENREF_11)] | M | OTH | CHERG (version a) |  |
| Black 2010 [[11](#_ENREF_11)] | ^b^ | NSI | CHERG (version b) |  |
| Bodnar 2014 [[12](#_ENREF_12)] | U |  |  | Dudley 2010-INCODE |
| Bonetti 2011 [[13](#_ENREF_13)] | U |  |  | Gardosi 2005-ReCoDe |
| Chan 2004 [[14](#_ENREF_14)] | O (m) | N | PSANZ-NDC |  |
| Chan 2004 [[14](#_ENREF_14)] | O (m) | M | PSANZ-PDC |  |
| CMACE 2010 [[15](#_ENREF_15)] | M | M | Maternal & fetal (UK national) |  |
| CMACE 2010 [[15](#_ENREF_15)] | N | N | Neonatal (UK national) |  |
| CMACE 2011 [[16](#_ENREF_16)] | M, U | U | Maternal & fetal (UK national) | CMACE 2010-neonatal |
| Cockerill 2012 [[17](#_ENREF_17)] | U |  |  | Gardosi 2005-ReCoDe |
| Cole 1986 [[18](#_ENREF_18)] | O (m) | M |  |  |
| Cole 1989 [[19](#_ENREF_19)] | O (m) | M | ICE |  |
| Cooke 2012 [[20](#_ENREF_20)] | U |  |  | Flenady 2009-PSANZ-NDC, Flenady 2009-PSANZ-PDC |
| Corcoran 2014 [[21](#_ENREF_21)] | U |  |  | Manning 2013-maternal & fetal-Ireland |
| Cunningham 1997^a^ [[22](#_ENREF_22)] | O | NSI |  |  |
| De Galan-Roosen 2002 [[23](#_ENREF_23)] | O (m), T | N | Fundamental Classification System |  |
| De Reu 2009 [[24](#_ENREF_24)] | M | U | Wigglesworth mod. |  |
| De Reu 2009 [[24](#_ENREF_24)] | M | U | Cole 1986 mod. |  |
| De Reu 2009 [[24](#_ENREF_24)] | M | U | Tulip mod. |  |
| De Reu 2011 [[25](#_ENREF_25)] | U |  |  | De Reu 2009-Tulip mod. |
| Dias e Silva 2013 [[26](#_ENREF_26)] | ^b^ | U | Brazilian list of avoidable deaths (Brazil national) |  |
| Doyle 2012 [[27](#_ENREF_27)] | T |  |  |  |
| Dudley 2010 [[28](#_ENREF_28)] | N | N | INCODE |  |
| Ecevit 2012 [[29](#_ENREF_29)] | U |  |  | Wigglesworth 1980 |
| Ego 2013 [[30](#_ENREF_30)] | U |  |  | Gardosi 2005-ReCoDe |
| Ellis 2011 [[31](#_ENREF_31)] | U |  |  | Wigglesworth 1980 |
| Engmann 2012 [[32](#_ENREF_32)] | M | U |  |  |
| Farquharson 2011 [[33](#_ENREF_33)] | U |  |  | CMACE 2010-maternal & fetal, CMACE 2010-neonatal, Wigglesworth 1980 |
| Fatima 2014 [[34](#_ENREF_34)] | U |  |  | Cunningham 1997 |
| Finn 2014 [[35](#_ENREF_35)] | U |  |  | Wigglesworth 1980 |
| Fleming 2009 [[36](#_ENREF_36)] | U |  |  | Wigglesworth 1980 |
| Flenady 2009 [[37](#_ENREF_37)] | T |  |  |  |
| Flenady 2009 [[38](#_ENREF_38)] | M | M | PSANZ-PDC (Australia and New Zealand national) |  |
| Flenady 2009 [[38](#_ENREF_38)] | M | M | PSANZ-NDC (Australia and New Zealand national) |  |
| Freitag 2014 [[39](#_ENREF_39)] | U |  |  | Gardosi 2005-ReCoDe |
| Freitas 2012 [[40](#_ENREF_40)] | ^b^ | U |  |  |
| Frøen 2009 [[41](#_ENREF_41)] | N, T | N | Codac |  |
| Frøen 2009 [[41](#_ENREF_41)] | M | M | Simplified Codac |  |
| Gardosi 2005 [[42](#_ENREF_42)] | O (n) | N | ReCoDe |  |
| Gardosi 2010 [[43](#_ENREF_43)] | U |  |  | Gardosi 2005-ReCoDe |
| Gardosi 2014^c^ | N, T | N | MAIN |  |
| Glinianaia 2010 [[44](#_ENREF_44)] | M | U |  |  |
| Gordijn 2009 [[45](#_ENREF_45)] | M | OTH | Multilayered approach |  |
| Gordon 2013 [[46](#_ENREF_46)] | U |  |  | Flenady 2009-PSANZ-PDC |
| Gupta 2012 [[47](#_ENREF_47)] | N, U | U | (Bhutan national) | Winter 2013-Rwanda |
| Hama Diallo 2012 [[48](#_ENREF_48)] | M | M |  |  |
| Headley 2009 [[49](#_ENREF_49)] | U |  |  | Flenady 2009-PSANZ-PDC |
| Heazell 2009 [[50](#_ENREF_50)] | U |  |  | Gardosi 2005-ReCoDe |
| Helgadottir 2013 [[51](#_ENREF_51)] | U |  |  | Frøen 2009-Codac |
| Hey 1986 [[52](#_ENREF_52)] | O (m) | M | Fetal and Neonatal Factors, short classification |  |
| Hinderaker 2003 [[53](#_ENREF_53)] | O (m) | U |  |  |
| Hirst 2012 [[54](#_ENREF_54)] | U, T |  |  | Flenady 2009-PSANZ-PDC |
| Holding 2014 [[55](#_ENREF_55)] | U |  |  | Gardosi 2005-ReCoDe |
| Ibiebele 2014 [[56](#_ENREF_56)] | U |  |  | Flenady 2009-PSANZ-PDC |
| Ibinabo 2013 [[57](#_ENREF_57)] | U |  |  | Flenady 2009-PSANZ-PDC |
| Jehan 2009^d^ [[58](#_ENREF_58)] | M | U |  |  |
| Kapurubandara 2011 [[59](#_ENREF_59)] | U |  |  | Flenady 2009-PSANZ-PDC |
| Kent 2009 [[60](#_ENREF_60)] | U |  |  | Chan 2004-PSANZ PDC & NDC |
| Kerridge 2013 [[61](#_ENREF_61)] | U |  |  | Flenady 2009-PSANZ-PDC |
| Khanal 2011 [[62](#_ENREF_62)] | ^b^ | U |  |  |
| Khanum 2009 [[63](#_ENREF_63)] | M | U |  |  |
| Kidanto 2009 [[64](#_ENREF_64)] | M, T | U |  |  |
| Kidron 2009 [[65](#_ENREF_65)] | M | U |  |  |
| Kinney 2010 [[66](#_ENREF_66)] | U |  |  | Lawn 2006-CHERG |
| Korkmaz 2010 [[67](#_ENREF_67)] | U |  |  | De Galan-Roosen 2002, Wigglesworth 1980 |
| Korteweg 2006 [[68](#_ENREF_68)] | O (n), T | N | Tulip |  |
| Korteweg 2009 [[69](#_ENREF_69)] | U |  |  | Korteweg 2006-Tulip |
| Korteweg 2010 [[70](#_ENREF_70)] | U |  |  | Korteweg 2006-Tulip |
| Korteweg 2012 [[71](#_ENREF_71)] | U |  |  | Korteweg 2006-Tulip |
| Kotecha 2014 [[72](#_ENREF_72)] | M, U | U | Clinico-pathological (Wales national) | Froen 2009-Codac, CMACE 2011-maternal & fetal |
| Kruse 2014 [[73](#_ENREF_73)] | M | U |  |  |
| Lawn 2006 [[74](#_ENREF_74)] | O (m) | OTH | CHERG |  |
| Lawn 2009 [[75](#_ENREF_75)] | U |  |  | Lawn 2006-CHERG |
| Lawn 2009 [[76](#_ENREF_76)] | N | OTH | Consistent Classification for Causes of Stillbirth |  |
| Lawn 2010 [[77](#_ENREF_77)] | M, U | U |  | Lawn 2006-CHERG, Black 2010-CHERG |
| Lawn 2012 [[78](#_ENREF_78)] | M, U | U |  | Black 2010-CHERG |
| Lawn 2014 [[79](#_ENREF_79)] | U |  |  | Black 2010-CHERG |
| Leite 2013 [[80](#_ENREF_80)] | U |  |  | Gardosi 2005-ReCoDe |
| Li 2013 [[81](#_ENREF_81)] | U |  |  | Flenady 2009-PSANZ-PDC |
| Liu 2012 [[82](#_ENREF_82)] | U |  |  | Black 2010-CHERG |
| Lu 2009 [[83](#_ENREF_83)] | U |  |  | Gardosi 2005-ReCoDe, Flenady 2009-PSANZ-PDC |
| Luo 2010 [[84](#_ENREF_84)] | U |  |  | Cole 1989-ICE |
| Manandhar 2010 [[85](#_ENREF_85)] | M | OTH |  |  |
| Manandhar 2011 [[86](#_ENREF_86)] | U |  |  | Wigglesworth 1980 |
| Manning 2013 [[87](#_ENREF_87)] | M, U | N | Maternal & fetal (Ireland national) | CMACE 2010-neonatal |
| Martins 2009 [[88](#_ENREF_88)] | U |  |  | Wigglesworth 1980 |
| McClure 2014^e^ [[89](#_ENREF_89)] | N | N | Global Network |  |
| Measey 2009 [[90](#_ENREF_90)] | U |  |  | Flenady 2009-PSANZ-PDC |
| Mmbaga 2012 [[91](#_ENREF_91)] | U |  |  | Winbo 1998-NICE |
| Mmbaga 2012 [[92](#_ENREF_92)] | U |  |  | Winbo 1998-NICE |
| Momena 2012 [[93](#_ENREF_93)] | U |  |  | Gardosi 2005-ReCoDe |
| Monari 2012 [[94](#_ENREF_94)] | U |  |  | Froen 2009-Codac |
| Mo-Suwan 2009 [[95](#_ENREF_95)] | M | M |  |  |
| MRC 2002 [[96](#_ENREF_96)] | O (m) | U | PPIP (South Africa national) |  |
| Nabeel 2012 [[97](#_ENREF_97)] | M | M |  |  |
| National Services Scotland 2013 [[98](#_ENREF_98)] | M | M | Obstetric (Scotland national) |  |
| National Services Scotland 2013 [[98](#_ENREF_98)] | ^b^ | U | FIGO (Scotland national) |  |
| National Services Scotland 2013 [[98](#_ENREF_98)] | M | M | Neonatal (Scotland national) |  |
| National Services Scotland 2014 [[99](#_ENREF_99)] | U |  |  | National Services Scotland 2013-obstetric, FIGO, & neonatal |
| Nausheen 2013 [[100](#_ENREF_100)] | M, T | M |  |  |
| Nga 2012 [[101](#_ENREF_101)] | M | U |  |  |
| Nijkamp 2013 [[102](#_ENREF_102)] | U |  |  | Korteweg 2006-Tulip |
| Nijkamp 2013 [[103](#_ENREF_103)] | U |  |  | Korteweg 2006-Tulip |
| NIPORT 2005 [[104](#_ENREF_104)] | O (m) | M | (Bangladesh national) |  |
| Olamijulo 2011 [[105](#_ENREF_105)] | M, U | U |  | Cole 1986 |
| Olegario 2013 [[106](#_ENREF_106)] | U |  |  | Hey 1986 |
| Oliva 2012 [[107](#_ENREF_107)] | U |  |  | Gardosi 2005-ReCoDe |
| Pattinson 1989 [[108](#_ENREF_108)] | O (m) | M |  |  |
| Pattinson 2014 [[109](#_ENREF_109)] | U |  |  | MRC 2002-PPIP |
| Perveen 2011 [[110](#_ENREF_110)] | U |  |  | Wigglesworth 1980 |
| PMMRC 2014 [[111](#_ENREF_111)] | U |  |  | Flenady 2009-PSANZ-PDC & NDC |
| Public Health Agency of Canada 2008 [[112](#_ENREF_112)] | O | U | (Canada national) |  |
| Public Health Agency of Canada 2013 [[113](#_ENREF_113)] | U |  |  | Cole 1989-ICE, Public Health Agency of Canada 2008 |
| Racape 2012 [[114](#_ENREF_114)] | U |  |  | Hey 1986-short form |
| Robalo 2013 [[115](#_ENREF_115)] | U |  |  | Gardosi 2005-ReCoDe |
| Rocha 2011 [[116](#_ENREF_116)] | ^b^ | U |  |  |
| Russo 2013 [[117](#_ENREF_117)] | U |  |  | Gardosi 2005-ReCoDe |
| Schmiegelow 2012 [[118](#_ENREF_118)] | M | M |  |  |
| Seaton 2012 [[119](#_ENREF_119)] | M, T | U |  |  |
| Serena 2013 [[120](#_ENREF_120)] | M | OTH | Aberdeen mod. |  |
| Serena 2013 [[120](#_ENREF_120)] | M | U | ReCoDe mod. |  |
| Shah 2011 [[121](#_ENREF_121)] | N | N |  |  |
| Shrestha 2010 [[122](#_ENREF_122)] | U |  |  | Wigglesworth 1980 |
| Shrestha 2012 [[123](#_ENREF_123)] | U |  |  | Wigglesworth 1980 |
| Simpson 2010 [[124](#_ENREF_124)] | M | M |  |  |
| Smith 2010 [[125](#_ENREF_125)] | M | M |  |  |
| The Stillbirth Collaborative Research Network Writing Group 2011 [[126](#_ENREF_126)] | M | U |  |  |
| Stormdal Bring 2014 [[127](#_ENREF_127)] | U |  |  | Varli 2008-Stockholm |
| Swenson 2014 [[128](#_ENREF_128)] | U |  |  | VanderWielen 2011-WiSSP |
| Talip 2010 [[129](#_ENREF_129)] | U |  |  | Pattinson 1989 |
| Tudehope 2013 [[130](#_ENREF_130)] | U |  |  | Flenady 2009-PSANZ-PDC & NDC |
| Ujwala 2012 [[131](#_ENREF_131)] | N, T | N |  |  |
| VanderWielen 2011 [[132](#_ENREF_132)] | N | OTH | WiSSP |  |
| Van Diem 2010 [[133](#_ENREF_133)] | M, U, T | U |  | Korteweg 2006-Tulip, Cole 1986 |
| Van Diem 2012 [[134](#_ENREF_134)] | U |  |  | Gordijn 2009 |
| Varli 2008 [[135](#_ENREF_135)] | O (n), T | N | Stockholm |  |
| Vieira 2012 [[136](#_ENREF_136)] | U |  |  | Gardosi 2005-ReCoDe |
| Waiswa 2010 [[137](#_ENREF_137)] | U |  |  | Lawn 2006-CHERG |
| Whitby 2014 [[138](#_ENREF_138)] | U |  |  | Gardosi 2005-ReCoDe |
| Wigglesworth 1980 [[139](#_ENREF_139)] | O (m) | N | Wigglesworth |  |
| Williams 2011 [[140](#_ENREF_140)] | U |  |  | Gardosi 2005-ReCoDe |
| Winbo 1998 [[141](#_ENREF_141)] | O (m), T | N | NICE |  |
| Winter 2013 [[142](#_ENREF_142)] | M | U | (Rwanda national) |  |
| Wood 2012 [[143](#_ENREF_143)] | M | M |  |  |
| Wou 2014 [[144](#_ENREF_144)] | M | U |  |  |
| Zhang 2009 [[145](#_ENREF_145)] | U |  |  | Cole 1989-ICE |

N=system newly created between 2009 and 2014, M=system modified between 2009 and 2014, O=older system (created/modified prior to 2009), m=older system that was modified prior to 2009, n=older system that was newly created prior to 2009, U=system is used, T=system is tested, NSI=no system intended, OTH=other intent, mod.=modification

^a^ System is described in the citation given, but originates from [[146](#_ENREF_146)].

^b^ Unknown whether new or modified; in most cases because we were unable to obtain the relevant document(s).

^c^ Personal communications, O. Tuncalp to V. Flenady, 7/21/2014 and 7/23/2014.

^d^ PubMed citation is for Imtiaz; we use the family name Jehan to refer to this system.

^e^ System was included via expert referral in 2014 and this paper was selected as the citation after its publication in 2015.

^f^ We judged that while the author intended to present a use of the ICD in order to validate a verbal autopsy tool, according to our definition, this is in fact a new system.

Note: “Other” intent: Gordijn 2009, Lawn 2009-Consistent classification for stillbirths, and VanderWielen 2011-WiSSP intended to create or modify not a "system for" classifying deaths per se, but an "approach to” classifying deaths; Black 2010 (CHERG a): intended to combine Lawn 2006 estimation approach with a new estimation approach; Lawn 2006 (CHERG): intended as a way to estimate global causes of NND, using a modification of existing systems; Manandhar 2010: intended to combine two systems (NICE and CHERG); Serena 2013 (Wigglesworth mod): intended as a combination of Wigglesworth and Aberdeen systems.

1. Abdellatif M, Al-Battashi A, Ahmed M, Bataclan MF, Khan AA, Al-Maniri A. The patterns and causes of neonatal mortality at a tertiary hospital in Oman. Oman Med J. 2013;28(6):422-6. doi:<http://dx.doi.org/10.5001/omj.2013.119>.

2. Abha S, Alpana T. Re. Co. De.: A better classification for determination of still births. Journal of Obstetrics and Gynecology of India. 2011;61(6):656-8.

3. Aggarwal AK, Jain V, Kumar R. Validity of verbal autopsy for ascertaining the causes of stillbirth. Bull World Health Organ. 2011;89(1):31-40. doi:10.2471/BLT.10.076828.

4. Aggarwal AK, Kumar P, Pandit S, Kumar R. Accuracy of WHO verbal autopsy tool in determining major causes of neonatal deaths in India. PLoS ONE [Electronic Resource]. 2013;8(1):e54865.

5. Akkhavong S. Infant Mortality and Maternal Mortality in Lao PDR. Training Course in Reproductive Health Research; 22 September 2009; Vientiane, Laos: GFMER, WHO, UNFPA, Lao PDR; 2009.

6. Aminu M, Unkels R, Mdegela M, Utz B, Adaji S, van den Broek N. Causes of and factors associated with stillbirth in low- and middle-income countries: a systematic literature review. BJOG. 2014;121 Suppl 4:141-53. doi:10.1111/1471-0528.12995.

7. Amouzou A, Richard SA, Friberg IK, Bryce J, Baqui AH, El Arifeen S et al. How well does LiST capture mortality by wealth quintile? A comparison of measured versus modelled mortality rates among children under-five in Bangladesh. Int J Epidemiol. 2010;39 Suppl 1:i186-92.

8. Andriamandimbison Z, Randriambololona DMA, Rasoanandrianina BS, Hery RA. Causes of in utero fetal deaths: 225 cases at Befelatanana Hospital Madagascar. [French]. Medecine et Sante Tropicales. 2013;23(1):78-82.

9. Bakketeig LS, Bergsjo P. Perinatal epidemiology. In: Van Look PFA, Heggenhougen HK, Quah SR, editors. Sexual and reproductive health: a public health perspective. Academic Press; 2011. p. 67-75.

10. Basys V, Drazdienë N, Vezbergienë N, Isakova J. Gimimø medicininiai duomenys [Medical data of Births 2013]. Vilnius. Institute of Hygiene Health Information Centre, Vilnius University Medical Faculty, Vilnius University, Centre of Neonatology; 2014.

11. Black RE, Cousens S, Johnson HL, Lawn JE, Rudan I, Bassani DG et al. Global, regional, and national causes of child mortality in 2008: a systematic analysis. Lancet. 2010;375(9730):1969-87. doi:10.1016/S0140-6736(10)60549-1.

12. Bodnar L, Parks WT, Perkins K, Abrams B, Feghali M, Pugh S et al. Prepregnancy obesity and the risk of cause-specific stillbirth. Am J Obstet Gynecol. 2014;210(1):S49.

13. Bonetti LR, Ferrari P, Trani N, Maccio L, Laura S, Giuliana S et al. The role of fetal autopsy and placental examination in the causes of fetal death: a retrospective study of 132 cases of stillbirths. Arch Gynecol Obstet. 2011;283(2):231-41.

14. Chan A, King JF, Flenady V, Haslam RH, Tudehope DI. Classification of perinatal deaths: development of the Australian and New Zealand classifications. J Paediatr Child Health. 2004;40(7):340-7. doi:10.1111/j.1440-1754.2004.00398.x.

15. Centre for Maternal and Child Enquiries (CMACE). Perinatal Mortality 2008: United Kingdom. London. CMACE; 2010.

16. Centre for Maternal and Child Enquiries (CMACE). Perinatal Mortality 2009: United Kingdom. London. CMACE; 2011.

17. Cockerill R, Whitworth MK, Heazell AEP. Do medical certificates of stillbirth provide accurate and useful information regarding the cause of death? Paediatr Perinat Epidemiol. 2012;26(2):117-23. doi:10.1111/j.1365-3016.2011.01247.x.

18. Cole SK, Hey EN, Thomson AM. Classifying perinatal death: an obstetric approach. Br J Obstet Gynaecol. 1986;93(12):1204-12.

19. Cole S, Hartford RB, Bergsjo P, McCarthy B. International collaborative effort (ICE) on birth weight, plurality, perinatal, and infant mortality. III: A method of grouping underlying causes of infant death to aid international comparisons. Acta Obstet Gynecol Scand. 1989;68(2):113-7.

20. Cooke L, Humphrey M, Mahomed K, VJ F. P153: Clinical scenarios in neonatal deaths in Queensland 1995-2008 using the PSANZ classification. FAOPS & PSANZ; Sydney, Australia; 2012.

21. Corcoran P, Manning E, Meaney S, Greene R. PPO.11 Perinatal mortality in Ireland: A national clinical audit. Archives of Disease in Childhood -- Fetal & Neonatal Edition. 2014;99:A153-A. doi:10.1136/archdischild-2014-306576.451.

22. Cunningham F LK, Bloom SL, Hauth JC, Rouse DJ, Spong CY, editor. Williams Obstetrics. 23rd ed. New York, NY: McGraw-Hill; 2010.

23. de Galan-Roosen AE, Kuijpers JC, van der Straaten PJ, Merkus JM. Fundamental classification of perinatal death. Validation of a new classification system of perinatal death. Eur J Obstet Gynecol Reprod Biol. 2002;103(1):30-6.

24. De Reu P, Van Diem M, Eskes M, Oosterbaan H, Smits L, Merkus H et al. The Dutch Perinatal Audit Project: a feasibility study for nationwide perinatal audit in the Netherlands. Acta Obstet Gynecol Scand. 2009;88(11):1201-8. doi:10.3109/00016340903280990.

25. De Reu PAOM, Oosterbaan HP, Smits LJM, Nijhuis JG. Perinatal mortality in preterm births: an analysis of causes, presence of substandard care and avoiding mortality in three Dutch regions. J Perinat Med. 2011;39(5):499-505. doi:10.1515/jpm.2011.064.

26. Dias e Silva CMC, Gomes KRO, Rocha OAMS, de Almeida IMLM, Neto JMM. Validity and reliability of data and avoidability of the underlying cause of neonatal deaths in the intensive care unit of the North-Northeast Perinatal Care Network [Validade, confiabilidade e evitabilidade da causa basica dos obitos neonatais ocorridos em unidade de cuidados intensivos da Rede Norte-Nordeste de Saude Perinatal]. Cad Saude Publica. 2013;29(3):547-56.

27. Doyle EM, Wishart V, Hennell C, Thornton CM. Stillbirth: Surely not 70% unexplained? Pediatr Dev Pathol. 2012;15 (5):417.

28. Dudley DJ, Goldenberg R, Conway D, Silver RM, Saade GR, Varner MW et al. A new system for determining the causes of stillbirth. Obstet Gynecol. 2010;116(2 PART 1):254-60.

29. Ecevit A, Oguz SS, Tarcan A, Yazici C, Dilmen U. The changing pattern of perinatal mortality and causes of death in central Anatolian region of Turkey. J Matern Fetal Neonatal Med. 2012;25(9):1738-41.

30. Ego A, Zeitlin J, Batailler P, Cornec S, Fondeur A, Baran-Marszak M et al. Stillbirth classification in population-based data and role of fetal growth restriction: the example of RECODE. BMC Pregnancy Childbirth. 2013;13(182).

31. Ellis M, Kishwar A, Biplob B, Shaha SK, Prost A, Rego AR et al. Intrapartum-related stillbirths and neonatal deaths in rural Bangladesh: a prospective, community-based cohort study. Pediatrics. 2011;127(5):e1182-e90. doi:10.1542/peds.2010-0842.

32. Engmann C, Garces A, Jehan I, Ditekemena J, Phiri M, Mazariegos M et al. Causes of community stillbirths and early neonatal deaths in low-income countries using verbal autopsy: an International, Multicenter Study. J Perinatol. 2012;32(8):585-92.

33. Farquharson SJ, Evans MJ, Denison FC, Stock SJ. The usefulness of blood tests for investigating cause of stillbirth: Do they provide additional information to postmortem and placental pathology? Arch Dis Child Fetal Neonatal Ed. 2011;96:Fa132-Fa3.

34. Fatima U, Sherwani R, Khan T, Zaheer S. Foetal autopsy-categories and causes of death. Journal of Clinical and Diagnostic Research. 2014;8(10):FC05-FC8.

35. Finn D, Collins A, Murphy BP, Dempsey EM. Mode of neonatal death in an Irish maternity centre. Eur J Pediatr. 2014;173(11):1505-9. doi:10.1007/s00431-014-2356-9.

36. Fleming P, Clarke T, Gormally SM. Irish neonatal mortality statistics for 2004 and over the past 17 years: how do we compare internationally? Ir Med J. 2009;102(4):111-3.

37. Flenady V, Froen JF, Pinar H, Torabi R, Saastad E, Guyon G et al. An evaluation of classification systems for stillbirth. BMC Pregnancy Childbirth. 2009;9:24.

38. Flenady V, King J, Charles A, Gardener G, Ellwood D, Day K et al. PSANZ Clinical Practice Guideline for Perinatal Mortality. Brisbane. Perinatal Society of Australia and New Zealand (PSANZ) Perinatal Mortality Group; 2009.

39. Freitag L, von Kaisenberg C, Kreipe HH, Hussein K. [Evaluation of intrauterine death: importance of examination of fetal, placental and maternal factors]. Pathologe. 2014;35(1):77-82. doi:10.1007/s00292-013-1858-y.

40. Freitas BAC, Goncalves MR, Ribeiro RdCL. Infant mortality according to preventable causes and components - Vicosa-MG, 1998-2010. [Portuguese]. Pediatria Moderna. 2012;48(6):237-45.

41. Froen JF, Pinar H, Flenady V, Bahrin S, Charles A, Chauke L et al. Causes of death and associated conditions (Codac) - a utilitarian approach to the classification of perinatal deaths. BMC Pregnancy Childbirth. 2009;9(22).

42. Gardosi J, Kady SM, McGeown P, Francis A, Tonks A. Classification of stillbirth by relevant condition at death (ReCoDe): population based cohort study. BMJ. 2005;331(7525):1113-7. doi:10.1136/bmj.38629.587639.7C.

43. Gardosi J, Francis A. Investigation of the clinical causes of stillbirth associated with maternal obesity. Arch Dis Child Fetal Neonatal Ed. 2010;95:Fa97.

44. Glinianaia SV, Rankin J, Pearce MS, Parker L, Pless-Mulloli T. Stillbirth and infant mortality in singletons by cause of death, birthweight, gestational age and birthweight-for-gestation, Newcastle upon Tyne 1961-2000. Paediatr Perinat Epidemiol. 2010;24(4):331-42. doi:10.1111/j.1365-3016.2010.01119.x.

45. Gordijn SJ, Korteweg FJ, Erwich JJHM, Holm JP, van Diem MT, Bergman KA et al. A multilayered approach for the analysis of perinatal mortality using different classification systems. Eur J Obstet Gynecol Reprod Biol. 2009;144(2):99-104.

46. Gordon A, Raynes-Greenow C, McGeechan K, Morris J, Jeffery H. Risk factors for antepartum stillbirth and the influence of maternal age in New South Wales Australia: a population based study. BMC Pregnancy Childbirth. 2013;13(12).

47. Gupta SS. Identification of causes of under-five deaths in health facilities in Bhutan Ministry of Health of the Royal Government of Bhutan 2012.

48. Hama Diallo A, Meda N, Sommerfelt H, Traore GS, Cousens S, Tylleskar T et al. The high burden of infant deaths in rural Burkina Faso: a prospective community-based cohort study. BMC Public Health. 2012;12:739.

49. Headley E, Gordon A, Jeffery H. Reclassification of unexplained stillbirths using clinical practice guidelines. Aust N Z J Obstet Gynaecol. 2009;49(3):285-9.

50. Heazell AE, Martindale EA. Can post-mortem examination of the placenta help determine the cause of stillbirth? J Obstet Gynaecol. 2009;29(3):225-8.

51. Helgadottir LB, Turowski G, Skjeldestad FE, Jacobsen AF, Sandset PM, Roald B et al. Classification of stillbirths and risk factors by cause of death - a case-control study. Acta Obstet Gynecol Scand. 2013;92(3):325-33. doi:10.1111/aogs.12044.

52. Hey EN, Lloyd DJ, Wigglesworth JS. Classifying perinatal death: fetal and neonatal factors. Br J Obstet Gynaecol. 1986;93(12):1213-23.

53. Hinderaker SG, Olsen BE, Bergsjo PB, Gasheka P, Lie RT, Havnen J et al. Avoidable stillbirths and neonatal deaths in rural Tanzania. BJOG. 2003;110(6):616-23.

54. Hirst JE, Ha LTT, Jeffery HE. Reducing the proportion of stillborn babies classified as unexplained in Vietnam by application of the PSANZ clinical practice guideline. Aust N Z J Obstet Gynaecol. 2012;52(1):62-6. doi:10.1111/j.1479-828X.2011.01363.x.

55. Holding G, Kenyon-Blair D, Spooner L, Collinge S, Mukherjee S, Satodia P. PPO.37Snapshot of Post-Mortem Examinations following Stillbirths at a University Hospital. Archives of Disease in Childhood -- Fetal & Neonatal Edition. 2014;99:A162-A. doi:10.1136/archdischild-2014-306576.477.

56. Ibiebele I, Coory M, Boyle F, Humphrey M, Vlack S, Flenady V. Stillbirth rates among indigenous and non-indigenous women in Queensland, Australia: is the gap closing? BJOG. 2014. doi:10.1111/1471-0528.13047.

57. Ibinabo I, Flenady V, Coory M, Boyle F, Charles A. Why so high? Unexplained stillbirths in Queensland, 1995-2004. J Paediatr Child Health. 2013;49:125.

58. Imtiaz J, Harris H, Sohail S, Amna Z, Naushaba M, Omrana P et al. Neonatal mortality, risk factors and causes: a prospective population-based cohort study in urban Pakistan. Bull World Health Organ. 2009;87(2):130-8. doi:10.2471/BLT.08.050963.

59. Kapurubandara S, Nayyar R, Mukherjee M, Jagadish U, Yim S, Alahakoon D et al. Westmead hospital perinatal mortality audit. J Paediatr Child Health. 2011;47:86.

60. Kent AL, Dahlstrom JE, Ellwood D, Bourne M, A. C. T. Perinatal Mortality Committee. Systematic multidisciplinary approach to reporting perinatal mortality: lessons from a five-year regional review. Aust N Z J Obstet Gynaecol. 2009;49(5):472-7.

61. Kerridge K, Wilson T, Cooke L, Flenady V. Improving accuracy in cause of perinatal death reporting through implementation of a perinatal bereavement service. J Paediatr Child Health. 2013;49:25.

62. Khanal S, GC VS, Dawson P, Houston R. Verbal autopsy to ascertain causes of neonatal deaths in a community setting: a study from Morang, Nepal. JNMA, Journal of the Nepal Medical Association. 2011;51(181):21-7.

63. Khanum F. Perinatal mortality-one year analysis at tertiary care hospital of Peshawar. Journal of Postgraduate Medical Institute. 2009;23(3):267-71.

64. Kidanto HL, Mogren I, van Roosmalen J, Thomas AN, Massawe SN, Nystrom L et al. Introduction of a qualitative perinatal audit at Muhimbili National Hospital, Dar es Salaam, Tanzania. BMC Pregnancy Childbirth. 2009;9:45.

65. Kidron D, Bernheim J, Aviram R. Placental findings contributing to fetal death, a study of 120 stillbirths between 23 and 40 weeks gestation. Placenta. 2009;30(8):700-4.

66. Kinney MV, Kerber KJ, Black RE, Cohen B, Nkrumah F, Coovadia H et al. Sub-Saharan Africa's mothers, newborns, and children: where and why do they die? PLoS Med. 2010;7(6). doi:10.1371/journal.pmed.1000294.

67. Korkmaz A, Akcoren Z, Alanay Y, Ozyuncu O, Yigit S, Deren O et al. Perinatal mortality analysis from 2001-2006 at Hacettepe University Hospital. [Turkish]. Cocuk Saglg ve Hastalklar Dergisi. 2010;53(3):175-88.

68. Korteweg FJ, Gordijn SJ, Timmer A, Erwich JJ, Bergman KA, Bouman K et al. The Tulip classification of perinatal mortality: introduction and multidisciplinary inter-rater agreement. BJOG. 2006;113(4):393-401. doi:10.1111/j.1471-0528.2006.00881.x.

69. Korteweg FJ, Erwich JJHM, Holm JP, Ravise JM, Van Der Meer J, Veeger NJGM et al. Diverse placental pathologies as the main causes of fetal death. Obstet Gynecol. 2009;114(4):809-17.

70. Korteweg FJ, Erwich JJ, Folkeringa N, Timmer A, Veeger NJ, Ravise JM et al. Prevalence of parental thrombophilic defects after fetal death and relation to cause. Obstet Gynecol. 2010;116(2 Pt 1):355-64. doi:10.1097/AOG.0b013e3181e66d58.

71. Korteweg FJ, Erwich JJHM, Timmer A, Van Der Meer J, Ravise JM, Veeger NJGM et al. Evaluation of 1025 fetal deaths: Proposed diagnostic workup. Am J Obstet Gynecol. 2012;206(1):53.e1-.e12.

72. Kotecha S, Kotecha S, Rolfe K, Barton E, John N, Lloyd M et al. All Wales Perinatal Survey Annual Report 2013 Cardiff, Wales; 2014.

73. Kruse AY, Phuong CN, Ho BTT, Stensballe LG, Pedersen FK, Greisen G. Identification of important and potentially avoidable risk factors in a prospective audit study of neonatal deaths in a paediatric hospital in Vietnam. Acta Paediatr. 2014;103(2):139-44. doi:10.1111/apa.12423.

74. Lawn JE, Wilczynska-Ketende K, Cousens SN. Estimating the causes of 4 million neonatal deaths in the year 2000. Int J Epidemiol. 2006;35(3):706-18. doi:10.1093/ije/dyl043.

75. Lawn JE, Lee ACC, Kinney M, Sibley L, Carlo WA, Paul VK et al. Two million intrapartum-related stillbirths and neonatal deaths: where, why, and what can be done? (Special Issue: Intrapartum-related deaths: evidence for action.). International Journal of Gynecology & Obstetrics. 2009;107(Suppl. 1):S5-S19. doi:10.1016/j.ijgo.2009.07.016.

76. Lawn JE, Yakoob MY, Haws RA, Tanya S, Darmstadt GL, Bhutta ZA. 3.2 million stillbirths: epidemiology and overview of the evidence review. (Special Issue: The global picture and evidence-based solutions.). BMC Pregnancy Childbirth. 2009;9(Suppl. 1). doi:10.1186/1471-2393-9-S1-S2.

77. Lawn JE, Kerber K, Enweronu-Laryea C, Cousens S. 3.6 Million neonatal deaths - what is progressing and what is not? (Special Issue: Global perinatal health.). Semin Perinatol. 2010;34(6):371-86. doi:10.1053/j.semperi.2010.09.011.

78. Lawn JE, Kinney MV, Black RE, Pitt C, Cousens S, Kerber K et al. Newborn survival: a multi-country analysis of a decade of change. (Special Issue: A decade of change for newborn survival, policy and programmes (2000-2010): A multi-country evaluation of progress towards scale.). Health Policy Plan. 2012;27(Suppl. 3). doi:10.1093/heapol/czs053.

79. Lawn JE, Blencowe H, Oza S, You D, Lee ACC, Waiswa P et al. Every newborn: Progress, priorities, and potential beyond survival. The Lancet. 2014;384(9938):189-205.

80. Leite TS, Ribeiro V, Leca O, De Castro Almeida D, Viana J, Lobo I. Stillbirths in a high income country: We can still make a difference. J Perinat Med. 2013;41(s1).

81. Li Z, Zeki R, Hilder L, Sullivan EA. Australia’s mothers and babies 2011. Canberra, Australia. AIHW National Perinatal Epidemiology and Statistics Unit; 2013.

82. Liu L, Johnson HL, Cousens S, Perin J, Scott S, Lawn JE et al. Global, regional, and national causes of child mortality: an updated systematic analysis for 2010 with time trends since 2000.[Erratum appears in Lancet. 2012 Oct 13;380(9850):1308]. Lancet. 2012;379(9832):2151-61.

83. Lu JR, McCowan L. A comparison of the Perinatal Society of Australia and New Zealand-Perinatal Death Classification system and relevant condition at death stillbirth classification systems. Aust N Z J Obstet Gynaecol. 2009;49(5):467-71.

84. Luo ZC, Senecal S, Simonet F, Guimond E, Penney C, Wilkins R. Birth outcomes in the Inuit-inhabited areas of Canada. CMAJ Canadian Medical Association Journal. 2010;182(3):235-42.

85. Manandhar SR, Ojha A, Manandhar DS, Shrestha B, Shrestha D, Saville N et al. Causes of stillbirths and neonatal deaths in Dhanusha district, Nepal: a verbal autopsy study. Kathmandu University Medical Journal. 2010;8(1):62-72.

86. Manandhar SR, Manandhar DS, Shrestha J, Karki C. Analysis of perinatal deaths and ascertaining perinatal mortality trend in a hospital. Journal of Nepal Health Research Council. 2011;9(2):150-3.

87. Manning E, Corcoran P, Meaney S, Greene RA, on behalf of the Perinatal Mortality Group. Perinatal Mortality in Ireland Annual Report 2011. Cork. National Perinatal Epidemiology Centre; 2013.

88. Martins EF, Rezende EM, Lana FCF. Causes and avoidability of perinatal deaths in Belo Horizonte, Minas Gerais [Portuguese]. Revista Mineira de Enfermagem. 2009;13(4):550-7.

89. McClure EM, Bose CL, Garces A, Esamai F, Goudar SS, Patel A et al. Global network for women's and children's health research: a system for low-resource areas to determine probable causes of stillbirth, neonatal, and maternal death. Maternal health, neonatology and perinatology. 2015;1:11. doi:10.1186/s40748-015-0012-7.

90. Measey MA, Tursan d'Espaignet E, Charles A, Douglass C. Unexplained fetal death: are women with a history of fetal loss at higher risk? Aust N Z J Obstet Gynaecol. 2009;49(2):151-7.

91. Mmbaga BT, Lie R, Olomi R, Mahande M, Olola O, Daltveit A. Causes of perinatal death at a tertiary care hospital in Northern Tanzania 2000-2010: a registry based study. BMC Pregnancy Childbirth. 2012;12(139).

92. Mmbaga BT, Lie RT, Olomi R, Mahande MJ, Kvale G, Daltveit AK. Cause-specific neonatal mortality in a neonatal care unit in Northern Tanzania: a registry based cohort study. BMC Pediatr. 2012;12:116. doi:10.1186/1471-2431-12-116.

93. Momena JA, Anita-Rao C. Three years study of perinatal mortality in a district general hospital, UK. BJOG. 2012;119:55.

94. Monari F, Alberico S, Avagliano L, Cetin I, Cozzolino S, Gargano G et al. Relation between maternal thrombophilia and stillbirth according to causes/associated conditions of death. Early Hum Dev. 2012;88(4):251-4.

95. Mo-suwan L, Isaranurug S, Chanvitan P, Techasena W, Sutra S, Supakunpinyo C et al. Perinatal death pattern in the four districts of Thailand: findings from the Prospective Cohort Study of Thai Children (PCTC). J Med Assoc Thai. 2009;92(5):660-6.

96. The MRC Unit for Maternal and Infant Health Care Strategies, PPIP Users, National Department of Health. Saving Babies 2002: Third Perinatal Care Survey of South Africa. 2002.

97. Nabeel M, Bushra M, Anum Y, Muneer A, Jai K. The study of etiological and demographic characteristics of neonatal mortality and morbidity - a consecutive case series study from Pakistan. BMC Pediatr. 2012;12(131).

98. National Services Scotland. Scottish Perinatal and Infant Mortality and Morbidity Report 2011. Edinburgh; 2013.

99. National Services Scotland. Scottish Perinatal and Infant Mortality and Morbidity Report 2012. Edinburgh; 2014.

100. Nausheen S, Soofi SB, Sadiq K, Habib A, Turab A, Memon Z et al. Validation of Verbal Autopsy Tool for Ascertaining the Causes of Stillbirth. PLoS One. 2013;8(10).

101. Nga NT, Hoa DT, Malqvist M, Persson LA, Ewald U. Causes of neonatal death: results from NeoKIP community-based trial in Quang Ninh province, Vietnam. Acta Paediatr. 2012;101(4):368-73.

102. Nijkamp J, Korteweg F, Groen H, Ravise J, Holm J, Timmer A et al. Maternal body mass index (BMI) and stillbirth: Analysis for potential causal pathophysiological mechanisms. Am J Obstet Gynecol. 2013;208 (1 SUPPL.1):S63.

103. Nijkamp JW, Korteweg FJ, Holm JP, Timmer A, Erwich JJHM, Van Pampus MG. Subsequent pregnancy outcome after previous foetal death. Eur J Obstet Gynecol Reprod Biol. 2013;166(1):37-42.

104. National Institute of Population Research and Training (NIPORT), Mitra and Associates, ORC Macro. Bangladesh Demographic and Health Survey 2004. Dhaka, Bangladesh, and Calverton, Maryland, USA. National Institute of Population Research and Training, Mitra and Associates, and ORC Macro; 2005.

105. Olamijulo JA, Olaleye O. Perinatal mortality in Lagos University Teaching Hospital: a five year review. Nig Q J Hosp Med. 2011;21(4):255-61.

106. Olegario JG, Silva MV, Machado JR, Rocha LP, Reis MA, Guimaraes CS et al. Pulmonary innate immune response and melatonin receptors in the perinatal stress. Clin Dev Immunol. 2013;2013:340959.

107. Oliva G, Leggieri C, D'Oria L, Casarella L, De Waure C, Di Nardo F et al. Epidemiological study on intrauterine fetal death: Analysis of 113 cases [Studio epidemiologico sulla morte endouterina fetale: Analisi di 113 casi]. Italian Journal of Gynaecology and Obstetrics. 2012;24(2):75-84.

108. Pattinson RC, De Jong G, Theron GB. Primary causes of total perinatally related wastage at Tygerberg Hospital. S Afr Med J. 1989;75(2):50-3.

109. Pattinson R, Rhoda N. Saving babies 2012-2013: Ninth report on perinatal care in South Africa. Pretoria, South Africa. Tshepesa Press; 2014.

110. Perveen F, Tayyab S, Zuberi BF. Risk factors for perinatal deaths in Pakistan. J Obstet Gynaecol Res. 2011;37(10):1359-64.

111. Perinatal and Maternal Mortality Review Committee (PMMRC). Eighth Annual Report of the Perinatal and Maternal Mortality Review Committee: Reporting Mortality 2012. Wellington, New Zealand. Health Quality & Safety Commission; 2014.

112. Public Health Agency of Canada. Canadian Perinatal Health Report, 2008 Edition. Ottawa, Canada; 2008.

113. Public Health Agency of Canada. Perinatal Health Indicators for Canada 2013: a Report of the Canadian Perinatal Surveillance System. Ottawa, Canada; 2013.

114. Racape J, Haelterman E, De Spiegelaere M, Dramaix M, Alexander S. Effect of adopting host-country nationality (naturalisation) on perinatal mortality rates and causes among immigrants in Brussels. J Matern Fetal Neonatal Med. 2012;25:44-5.

115. Robalo R, Pedroso C, Amaral N, Soares C. Late stillbirth: A ten year cohort study [Fetomortalidade tardia: Estudo coorte de dez anos]. Acta Med Port. 2013;26(1):39-42.

116. Rocha R, Oliveira C, Karina Ferreira D, Bonfim C. Neonatal mortality and avoidability: an epidemiological profile analysis [Portuguese]. Revista Enfermagem UERJ. 2011;19(1):114-20.

117. Russo FM, Pozzi E, Pelizzoni F, Todyrenchuk L, Bernasconi DP, Cozzolino S et al. Stillbirths in singletons, dichorionic and monochorionic twins: A comparison of risks and causes. Eur J Obstet Gynecol Reprod Biol. 2013;170(1):131-6.

118. Schmiegelow C, Minja D, Oesterholt M, Pehrson C, Suhrs HE, Bostrom S et al. Factors associated with and causes of perinatal mortality in northeastern Tanzania. Acta Obstet Gynecol Scand. 2012;91(9):1061-8.

119. Seaton SE, Field DJ, Draper ES, Manktelow BN, Smith GCS, Springett A et al. Socioeconomic inequalities in the rate of stillbirths by cause: A population-based study. BMJ Open. 2012;2(3).

120. Serena C, Marchetti G, Rambaldi MP, Ottanelli S, Di Tommaso M, Avagliano L et al. Stillbirth and fetal growth restriction. J Matern Fetal Neonatal Med. 2013;26(1):16-20.

121. Shah BD, Dwivedi LK. Causes of neonatal deaths among tribal women in Gujarat, India. Population Research and Policy Review. 2011;30(4):517-36. doi:10.1007/s11113-010-9199-5.

122. Shrestha S, Sharma A, Upadhyay S, Rijal P. Perinatal mortality audit. Nepal Medical College Journal: NMCJ. 2010;12(4):257-9.

123. Shrestha M, Shrestha L, Basnet S, Shrestha PS. Trends in perinatal mortality in Tribhuvan University Teaching Hospital: 13 years review. Journal of Nepal Paediatric Society. 2012;32(2):150-3.

124. Simpson CD, Ye XY, Hellmann J, Tomlinson C. Trends in cause-specific mortality at a Canadian outborn NICU. Pediatrics. 2010;126(6):e1538-44.

125. Smith LK, Manktelow BN, Draper ES, Springett A, Field DJ. Nature of socioeconomic inequalities in neonatal mortality: population based study. BMJ. 2010;341(c6654).

126. The Stillbirth Collaborative Research Network Writing Group. Causes of death among stillbirths. J Am Med Assoc. 2011;306(22):2459-68.

127. Stormdal Bring H, Hulthen Varli IA, Kublickas M, Papadogiannakis N, Pettersson K. Causes of stillbirth at different gestational ages in singleton pregnancies. Acta Obstet Gynecol Scand. 2014;93(1):86-92. doi:10.1111/aogs.12278.

128. Swenson E, Schema L, McPherson E. Radiographic evaluation of stillbirth: What does it contribute? Am J Med Genet A. 2014;164(9):2270-5.

129. Talip Q, Theron G, Steyn W, Hall D. Total perinatally related losses at Tygerberg Hospital - a comparison between 1986, 1993 and 2006. S Afr Med J. 2010;100(4):250-3.

130. Tudehope D, Papadimos E, Gibbons K. Twelve-year review of neonatal deaths in the delivery room in a perinatal tertiary centre. J Paediatr Child Health. 2013;49(1):E40-5.

131. Ujwala B, Alcock G, More NS, Sushmita D, Wasundhara J, Osrin D. Stillbirths and newborn deaths in slum settlements in Mumbai, India: a prospective verbal autopsy study. BMC Pregnancy Childbirth. 2012;12(39).

132. VanderWielen B, Zaleski C, Cold C, McPherson E. Wisconsin stillbirth services program: a multifocal approach to stillbirth analysis. Am J Med Genet A. 2011;155A(5):1073-80. doi:10.1002/ajmg.a.34016.

133. van Diem M, De Reu P, Eskes M, Brouwers H, Holleboom C, Slagter-Roukema T et al. National perinatal audit, a feasible initiative for the Netherlands!? A validation study. Acta Obstet Gynecol Scand. 2010;89(9):1168-73.

134. van Diem MT, Timmer A, Bergman KA, Bouman K, van Egmond N, Stant DA et al. The implementation of unit-based perinatal mortality audit in perinatal cooperation units in the northern region of the Netherlands. BMC Health Serv Res. 2012;12:195. doi:10.1186/1472-6963-12-195.

135. Varli IH, Petersson K, Bottinga R, Bremme K, Hofsjo A, Holm M et al. The Stockholm classification of stillbirth. Acta Obstet Gynecol Scand. 2008;87(11):1202-12. doi:10.1080/00016340802460271.

136. Vieira MSM, Siebert EC, Ceglio WQGW, De Almeira MH, Batista TS, Freitas PF. Difficulties for identification of cause of fetal death: how to solve? [Portuguese]. Revista Brasileira de Ginecologia e Obstetricia. 2012;34(9):403-8. doi:10.1590/S0100-72032012000900003.

137. Waiswa P, Kallander K, Peterson S, Tomson G, Pariyo GW. Using the three delays model to understand why newborn babies die in eastern Uganda. Trop Med Int Health. 2010;15(8):964-72. doi:10.1111/j.1365-3156.2010.02557.x.

138. Whitby EH, Offiah A, Cohen MC. Initial experiences of a minimally invasive autopsy service. Pediatr Radiol. 2014;44:S324.

139. Wigglesworth JS. Monitoring perinatal mortality. A pathophysiological approach. Lancet. 1980;2(8196):684-6.

140. Williams M, El-Sheikh A, Malick A, Gardosi J. Maternal age and risk of stillbirth. Arch Dis Child Fetal Neonatal Ed. 2011;96:Fa2.

141. Winbo IG, Serenius FH, Dahlquist GG, Kallen BA. NICE, a new cause of death classification for stillbirths and neonatal deaths. Neonatal and Intrauterine Death Classification according to Etiology. Int J Epidemiol. 1998;27(3):499-504.

142. Winter R, Pullum T, Langston A, Mivumbi NV, Rutayisire PC, Muhoza DN et al. Trends in Neonatal Mortality in Rwanda, 2000-2010. Calverton, Maryland, USA. ICF International; 2013.

143. Wood AM, Pasupathy D, Pell JP, Fleming MS. Trends in socioeconomic inequalities in risk of sudden infant death syndrome, other causes of infant mortality, and stillbirth in Scotland: population based study. BMJ: British Medical Journal (Overseas & Retired Doctors Edition). 2012;344(7850):21-. doi:10.1136/bmj.e1552.

144. Wou K, Ouellet MP, Chen MF, Brown RN. Comparison of the aetiology of stillbirth over five decades in a single centre: A retrospective study. BMJ Open. 2014;4(6).

145. Zhang X, Kramer MS. Variations in mortality and morbidity by gestational age among infants born at term. J Pediatr. 2009;154(3):358-62. doi:10.1016/j.jpeds.2008.09.013.

146. Cunningham FG, Hollier LM. Fetal death. In: Williams Obstetrics. 20th ed (Suppl 4) ed. Norwalk, Conn.: Appleton & Lange; August/September 1997.
