# Supplementary material for: Seeking order amidst chaos: a systematic review of classification systems for causes of stillbirth and neonatal death, 2009–2014
Source: BMC Pregnancy Childbirth. 2016 Oct 5;16:295. doi: 10.1186/s12884-016-1071-0 (PMC5053068; doi:10.1186/s12884-016-1071-0)
Supplement: Additional file 6: — Year of creation/modification of classification systems for causes of stillbirth and neonatal death developed or used between 2009 and 2014. (DOCX 48 kb) [file 12884_2016_1071_MOESM6_ESM.docx]

## Additional file 6

### Year of creation/modification of classification systems for causes of stillbirth and neonatal death developed or used between 2009 and 2014
