# Supplementary material for: Seeking order amidst chaos: a systematic review of classification systems for causes of stillbirth and neonatal death, 2009–2014
Source: BMC Pregnancy Childbirth. 2016 Oct 5;16:295. doi: 10.1186/s12884-016-1071-0 (PMC5053068; doi:10.1186/s12884-016-1071-0)
Supplement: Additional file 7: — Countries in which more than one system was used to classify causes of stillbirths and/or neonatal deaths, 2009-2014. (DOCX 45 kb) [file 12884_2016_1071_MOESM7_ESM.docx]

## Additional file 7

### Countries in which more than one system was used to classify causes of stillbirths and/or neonatal deaths, 2009-2014

| Country | # systems used | # systems used that are national |
| --- | --- | --- |
| UK | 8 | 3 |
| Netherlands | 7 | - |
| Brazil | 6 | 1 |
| India | 6 | - |
| Pakistan | 6 | - |
| Canada | 5 | 1 |
| Australia | 4 | 2 |
| Italy | 4 | - |
| Scotland | 4 | 3 |
| Tanzania | 4 | - |
| USA | 4 | - |
| Ireland | 3 | 1 |
| Nepal | 3 | - |
| New Zealand | 3 | 1 |
| Vietnam | 3 | - |
| Wales | 3 | 1 |
| Bangladesh | 2 | 1 |
| Bhutan | 2 | 1 |
| Nigeria | 2 | - |
| South Africa | 2 | 1 |
| Turkey | 2 | - |
