# Supplementary material for: Seeking order amidst chaos: a systematic review of classification systems for causes of stillbirth and neonatal death, 2009–2014
Source: BMC Pregnancy Childbirth. 2016 Oct 5;16:295. doi: 10.1186/s12884-016-1071-0 (PMC5053068; doi:10.1186/s12884-016-1071-0)
Supplement: Additional file 8: — National classification systems for causes of stillbirth and neonatal death in use 2009-2014. (DOCX 53 kb) [file 12884_2016_1071_MOESM8_ESM.docx]

## Additional file 8

### National classification systems for causes of stillbirth and neonatal death in use 2009-2014

| **High-income country national systems**   1. Australia, New Zealand: Flenady 2009-PSANZ-NDC [[1](#_ENREF_1)] 2. Australia, New Zealand: Flenady 2009-PSANZ-PDC [[1](#_ENREF_1)] 3. Canada: Public Health Agency of Canada 2008 [[2](#_ENREF_2)] 4. Ireland: Manning 2013-maternal & fetal [[3](#_ENREF_3)] 5. Lithuania: Basys 2014 [[4](#_ENREF_4)] 6. Scotland: National Services Scotland 2013-FIGO [[5](#_ENREF_5)] 7. Scotland: National Services Scotland 2013-neonatal [[5](#_ENREF_5)] 8. Scotland: National Services Scotland 2013-obstetric [[5](#_ENREF_5)] 9. UK: CMACE 2010-maternal & fetal [[6](#_ENREF_6)] 10. UK, Wales: CMACE 2011-maternal & fetal [[7](#_ENREF_7)] 11. UK, Ireland: CMACE 2010-neonatal [[6](#_ENREF_6)] 12. Wales: Kotecha 2014 [[8](#_ENREF_8)]   **Low- and middle-income country national systems**   1. Bangladesh: NIPORT 2005 [[9](#_ENREF_9)] 2. Bhutan: Gupta 2012 [[10](#_ENREF_10)] 3. Brazil: Dias e Silva 2013-Brazilian List of Avoidable Deaths [[11](#_ENREF_11)] 4. Rwanda, Bhutan: Winter 2013 [[12](#_ENREF_12)] 5. South Africa: MRC 2002-PPIP [[13](#_ENREF_13)] |
| --- |

1. Flenady V, King J, Charles A, Gardener G, Ellwood D, Day K et al. PSANZ Clinical Practice Guideline for Perinatal Mortality. Brisbane. Perinatal Society of Australia and New Zealand (PSANZ) Perinatal Mortality Group; 2009.

2. Public Health Agency of Canada. Canadian Perinatal Health Report, 2008 Edition. Ottawa, Canada; 2008.

3. Manning E, Corcoran P, Meaney S, Greene RA, on behalf of the Perinatal Mortality Group. Perinatal Mortality in Ireland Annual Report 2011. Cork. National Perinatal Epidemiology Centre; 2013.

4. Basys V, Drazdienë N, Vezbergienë N, Isakova J. Gimimø medicininiai duomenys [Medical data of Births 2013]. Vilnius. Institute of Hygiene Health Information Centre, Vilnius University Medical Faculty, Vilnius University, Centre of Neonatology; 2014.

5. National Services Scotland. Scottish Perinatal and Infant Mortality and Morbidity Report 2011. Edinburgh; 2013.

6. Centre for Maternal and Child Enquiries (CMACE). Perinatal Mortality 2008: United Kingdom. London. CMACE; 2010.

7. Centre for Maternal and Child Enquiries (CMACE). Perinatal Mortality 2009: United Kingdom. London. CMACE; 2011.

8. Kotecha S, Kotecha S, Rolfe K, Barton E, John N, Lloyd M et al. All Wales Perinatal Survey Annual Report 2013 Cardiff, Wales; 2014.

9. National Institute of Population Research and Training (NIPORT), Mitra and Associates, ORC Macro. Bangladesh Demographic and Health Survey 2004. Dhaka, Bangladesh, and Calverton, Maryland, USA. National Institute of Population Research and Training, Mitra and Associates, and ORC Macro; 2005.

10. Gupta SS. Identification of causes of under-five deaths in health facilities in Bhutan Ministry of Health of the Royal Government of Bhutan 2012.

11. Dias e Silva CMC, Gomes KRO, Rocha OAMS, de Almeida IMLM, Neto JMM. Validity and reliability of data and avoidability of the underlying cause of neonatal deaths in the intensive care unit of the North-Northeast Perinatal Care Network [Validade, confiabilidade e evitabilidade da causa basica dos obitos neonatais ocorridos em unidade de cuidados intensivos da Rede Norte-Nordeste de Saude Perinatal]. Cad Saude Publica. 2013;29(3):547-56.

12. Winter R, Pullum T, Langston A, Mivumbi NV, Rutayisire PC, Muhoza DN et al. Trends in Neonatal Mortality in Rwanda, 2000-2010. Calverton, Maryland, USA. ICF International; 2013.

13. The MRC Unit for Maternal and Infant Health Care Strategies, PPIP Users, National Department of Health. Saving Babies 2002: Third Perinatal Care Survey of South Africa. 2002.
