# Supplementary material for: Seeking order amidst chaos: a systematic review of classification systems for causes of stillbirth and neonatal death, 2009–2014
Source: BMC Pregnancy Childbirth. 2016 Oct 5;16:295. doi: 10.1186/s12884-016-1071-0 (PMC5053068; doi:10.1186/s12884-016-1071-0)
Supplement: Additional file 9: — Classification systems for causes of stillbirth and neonatal death used in highest-burden countries, 2009-2014. (DOCX 83 kb) [file 12884_2016_1071_MOESM9_ESM.docx]

## Additional file 9

### Classification systems for causes of stillbirth and neonatal death used in highest-burden countries, 2009-2014

| Country | # deaths | Systems used |
| --- | --- | --- |
| Countries with highest neonatal death burden, 2015 numbers [[1](#_ENREF_1)] | | |
| 1. India | 696,000 | Ujwala 2012 [[2](#_ENREF_2)]; Shah 2011 [[3](#_ENREF_3)]; Aggarwal 2013 [[4](#_ENREF_4)] |
| 1. Pakistan | 245,000 | Engmann 2012 [[5](#_ENREF_5)]; Jehan 2009 [[6](#_ENREF_6)]; Khanum 2009 [[7](#_ENREF_7)]; Nabeel 2012 [[8](#_ENREF_8)]; Wigglesworth 1980 [[9](#_ENREF_9)] |
| 1. Nigeria | 240,000 | Cole 1986 [[10](#_ENREF_10)]; Olamijulo 2011 [[11](#_ENREF_11)] |
| 1. DRC | 94,000 | Engmann 2012 [[5](#_ENREF_5)] |
| 1. China | 93,000 | none |
| 1. Ethiopia | 87,000 | none |
| 1. Bangladesh | 74,000 | NIPORT 2005-Bangladesh [[12](#_ENREF_12)]; Wigglesworth 1980 [[9](#_ENREF_9)] |
| 1. Indonesia | 74,000 | none |
| 1. Angola | 53,000 | none |
| 1. Sudan | 39,000 | none |
| 1. Tanzania | 39,000 | Hinderaker 2003 [[13](#_ENREF_13)]; Kidanto 2009 [[14](#_ENREF_14)]; Schmiegelow 2012 [[15](#_ENREF_15)]; Winbo 1998-NICE [[16](#_ENREF_16)] |
| total | 1,734,000 |  |
| Countries with highest stillbirth burden, 2009 numbers [[17](#_ENREF_17)] | | |
| 1. India | 605,000 | Ujwala 2012 [[2](#_ENREF_2)]; Cunningham 1997 [[18](#_ENREF_18)]; Abha 2011 [[19](#_ENREF_19)]; Aggarwal 2011 [[20](#_ENREF_20)] |
| 1. Pakistan | 265,000 | Engmann 2012 [[5](#_ENREF_5)]; Khanum 2009 [[7](#_ENREF_7)]; Nausheen 2013 [[21](#_ENREF_21)]; Wigglesworth 1980 [[9](#_ENREF_9)] |
| 1. Nigeria | 264,000 | Cole 1986 [[10](#_ENREF_10)]; Olamijulo 2011 [[11](#_ENREF_11)] |
| 1. China | 182,000 | none |
| 1. Bangladesh | 129,000 | Wigglesworth 1980 [[9](#_ENREF_9)] |
| 1. DRC | 86,000 | Engmann 2012 [[5](#_ENREF_5)] |
| 1. Ethiopia | 82,000 | none |
| 1. Indonesia | 62,000 | none |
| 1. Tanzania | 48,000 | Hinderaker 2003 [[13](#_ENREF_13)]; Kidanto 2009 [[14](#_ENREF_14)]; Schmiegelow 2012 [[15](#_ENREF_15)]; Winbo 1998-NICE [[16](#_ENREF_16)] |
| 1. Afghanistan | 39,000 | none |
| total | 1,762,000 |  |

Table excludes the ICD and systems that estimate global causes; stillbirth numbers rounded to nearest thousand.

1. You D, Hug L, Ejdemyr S, Beise J, on behalf of the United Nations Inter-agency Group for Child Mortality Estimation (UN IGME). Levels and Trends in Child Mortality Report 2015. New York. United Nations Children’s Fund; 2015.

2. Ujwala B, Alcock G, More NS, Sushmita D, Wasundhara J, Osrin D. Stillbirths and newborn deaths in slum settlements in Mumbai, India: a prospective verbal autopsy study. BMC Pregnancy Childbirth. 2012;12(39).

3. Shah BD, Dwivedi LK. Causes of neonatal deaths among tribal women in Gujarat, India. Population Research and Policy Review. 2011;30(4):517-36. doi:10.1007/s11113-010-9199-5.

4. Aggarwal AK, Kumar P, Pandit S, Kumar R. Accuracy of WHO verbal autopsy tool in determining major causes of neonatal deaths in India. PLoS ONE [Electronic Resource]. 2013;8(1):e54865.

5. Engmann C, Garces A, Jehan I, Ditekemena J, Phiri M, Mazariegos M et al. Causes of community stillbirths and early neonatal deaths in low-income countries using verbal autopsy: an International, Multicenter Study. J Perinatol. 2012;32(8):585-92.

6. Imtiaz J, Harris H, Sohail S, Amna Z, Naushaba M, Omrana P et al. Neonatal mortality, risk factors and causes: a prospective population-based cohort study in urban Pakistan. Bull World Health Organ. 2009;87(2):130-8. doi:10.2471/BLT.08.050963.

7. Khanum F. Perinatal mortality-one year analysis at tertiary care hospital of Peshawar. Journal of Postgraduate Medical Institute. 2009;23(3):267-71.

8. Nabeel M, Bushra M, Anum Y, Muneer A, Jai K. The study of etiological and demographic characteristics of neonatal mortality and morbidity - a consecutive case series study from Pakistan. BMC Pediatr. 2012;12(131).

9. Wigglesworth JS. Monitoring perinatal mortality. A pathophysiological approach. Lancet. 1980;2(8196):684-6.

10. Cole SK, Hey EN, Thomson AM. Classifying perinatal death: an obstetric approach. Br J Obstet Gynaecol. 1986;93(12):1204-12.

11. Olamijulo JA, Olaleye O. Perinatal mortality in Lagos University Teaching Hospital: a five year review. Nig Q J Hosp Med. 2011;21(4):255-61.

12. National Institute of Population Research and Training (NIPORT), Mitra and Associates, ORC Macro. Bangladesh Demographic and Health Survey 2004. Dhaka, Bangladesh, and Calverton, Maryland, USA. National Institute of Population Research and Training, Mitra and Associates, and ORC Macro; 2005.

13. Hinderaker SG, Olsen BE, Bergsjo PB, Gasheka P, Lie RT, Havnen J et al. Avoidable stillbirths and neonatal deaths in rural Tanzania. BJOG. 2003;110(6):616-23.

14. Kidanto HL, Mogren I, van Roosmalen J, Thomas AN, Massawe SN, Nystrom L et al. Introduction of a qualitative perinatal audit at Muhimbili National Hospital, Dar es Salaam, Tanzania. BMC Pregnancy Childbirth. 2009;9:45.

15. Schmiegelow C, Minja D, Oesterholt M, Pehrson C, Suhrs HE, Bostrom S et al. Factors associated with and causes of perinatal mortality in northeastern Tanzania. Acta Obstet Gynecol Scand. 2012;91(9):1061-8.

16. Winbo IG, Serenius FH, Dahlquist GG, Kallen BA. NICE, a new cause of death classification for stillbirths and neonatal deaths. Neonatal and Intrauterine Death Classification according to Etiology. Int J Epidemiol. 1998;27(3):499-504.

17. Lawn JE, Blencowe H, Waiswa P, Amouzou A, Mathers C, Hogan D et al. Stillbirths: rates, risk factors, and acceleration towards 2030. The Lancet. 2016;387(10018):587-603.

18. Cunningham F LK, Bloom SL, Hauth JC, Rouse DJ, Spong CY, editor. Williams Obstetrics. 23rd ed. New York, NY: McGraw-Hill; 2010.

19. Abha S, Alpana T. Re. Co. De.: A better classification for determination of still births. Journal of Obstetrics and Gynecology of India. 2011;61(6):656-8.

20. Aggarwal AK, Jain V, Kumar R. Validity of verbal autopsy for ascertaining the causes of stillbirth. Bull World Health Organ. 2011;89(1):31-40. doi:10.2471/BLT.10.076828.

21. Nausheen S, Soofi SB, Sadiq K, Habib A, Turab A, Memon Z et al. Validation of Verbal Autopsy Tool for Ascertaining the Causes of Stillbirth. PLoS One. 2013;8(10).
