# Supplementary material for: Seeking order amidst chaos: a systematic review of classification systems for causes of stillbirth and neonatal death, 2009–2014
Source: BMC Pregnancy Childbirth. 2016 Oct 5;16:295. doi: 10.1186/s12884-016-1071-0 (PMC5053068; doi:10.1186/s12884-016-1071-0)
Supplement: Additional file 10: — Maximum percent of deaths classified as “other” and “unexplained” by classification systems according to type of death classified. (DOCX 62 kb) [file 12884_2016_1071_MOESM10_ESM.docx]

## Additional file 10

### Maximum percent of deaths classified as “other” and “unexplained” by classification systems according to type of death classified

#### Figure 9.1: Maximum percent of deaths classified as "other" by classification systems that only include stillbirths, 2009-2014

#### Figure 9.2: Maximum percent of deaths classified as "other" by classification systems that only include neonatal deaths, 2009-2014

#### Figure 9.3: Maximum percent of deaths classified as "other" by classification systems that include both stillbirths and neonatal deaths, 2009-2014

Note: The following systems had no “other” category: CMACE 2010-maternal & fetal, CMACE 2011 maternal & fetal, Flenady 2009-PSANZ-PDC, Gardosi 2005-ReCoDe, Lawn 2010, National Services Scotland 2013-FIGO, Rocha 2011, and Wood 2012. Dudley 2010-INCODE and Lawn 2012 had “other” categories but no data were available to report.

Categories that we interpreted as “other” in the 81 systems are the same as those reported in a companion paper in this series (Reinebrant H, Zheyi T, Wojcieszek AM, Coory M, Gardener G, Lourie R et al: Causes of stillbirth globally – burden in high- and low-resource settings, in preparation), and include: accident and other specific causes, acute antepartum event, conditions specific to the neonate, due to other specific causes, miscellaneous, miscellaneous/other specific causes, obstetric complications, obstetric problems, other, other antepartum-specified, other causes, other causes/unclassifiable, other conditions, other neonatal disorders, other or unknown, other or unspecified cause, other pertinent condition not specified, other specific causes, other specific conditions, other sudden or unexplained infant death, post-maturity, pregnancy-related, special causes, special conditions, specific, specific causes, specific conditions, specific diagnosis, and termination.

#### Figure 9.4: Maximum percent of deaths classified as "unexplained" by classification systems that only include stillbirths, 2009-2014

#### Figure 9.5: Maximum percent of deaths classified as "unexplained" by classification systems that only include neonatal deaths, 2009-2014

#### Figure 9.6: Maximum percent of deaths classified as "unexplained" by classification systems that include both stillbirths and neonatal deaths, 2009-2014

Note: The following systems had no “unexplained” category: Flenady 2009-PSANZ-NDC, Lawn

2012, Lawn 2010, and Winter 2013-Rwanda. Dudley 2010-INCODE had such a category but there were no data available to report.

Categories that we interpreted as “unexplained” in the 81 systems are the same as those reported in a companion paper in this series (Reinebrant H, Zheyi T, Wojcieszek AM, Coory M, Gardener G, Lourie R et al: Causes of stillbirth globally – burden in high- and low-resource settings, in preparation), and include: anoxia, antenatal death, antepartum (macerated), antepartum hypoxia, antepartum SB, asphyxia, asphyxia and unexplained deaths prior to onset of labour, asphyxia not explained by any maternal condition, asphyxia-related conditions, associated obstetric factors, associated with obstetric complication, cause not identified, cause undetermined due to insufficient information, cause unknown, early neonatal death, unknown/no cause, idiopathic, idiopathic/unknown etiology, inconclusive, intrauterine death, intrauterine hypoxia/asphyxia, IUFD, macerated, macerated fetus, missing, NND weighing 1000g+ and normally formed, NND weighing 500g+, no antecedent or associated obstetric factors, no AP condition identified, no consensus, no identified cause of death, no information, no information available, no NN condition identified, no obstetric antecedent, no obstetric cause, no obstetric cause/not applicable, no relevant condition identified, non-determined etiology, normally formed macerated SB, not assessed, SB weighing 1000g+ and normally formed, SB weighing 500g+, unknown/no cause, SIDS, sudden death, sudden infant death, sudden unexpected death, SUDI, time of SB unknown, totally unexplained, unable to classify, unable to classify due to lack of information, “unavoidable, partially avoidable and undefined”, unclassifiable, unclassified, unclassified-no relevant condition identified, unclassified/unknown/unexplained, undefined, undefined deaths, unexplained, unexplained <2.5 kg, unexplained >2.5kg, unexplained antepartum death, unexplained antepartum stillbirth <37 weeks, unexplained antepartum stillbirth ≥37 weeks, unexplained asphyxia, unexplained IUD, unexplained preterm birth (< 37 weeks gestation), unexplained stillbirth, unknown, unknown antepartum (not SGA), unknown cause, “unknown, unexplained, & unclassifiable”, and unknown/unexplained.

NOTE: We reported only the *maximum* percent of deaths classified as “other” and “unexplained” as it was felt this could best provide insight into the contribution of categories which are unhelpful to the prevention of stillbirth and neonatal deaths as they provide minimal or no insight into the factors at play. The terms “other” and “unexplained”, and the many terms that we included in these broad categories, most likely mean different things in different systems; definitions of these terms are few, and sometimes overlap. We included only data from top-level “other” and “unexplained” categories. Where a term included words belonging to both “other” and “unexplained” groups, we put it with the group associated with the first word in the term.
