# Supplementary material for: Seeking order amidst chaos: a systematic review of classification systems for causes of stillbirth and neonatal death, 2009–2014
Source: BMC Pregnancy Childbirth. 2016 Oct 5;16:295. doi: 10.1186/s12884-016-1071-0 (PMC5053068; doi:10.1186/s12884-016-1071-0)
Supplement: Additional file 11: — Kappa scores from reliability testing of classification systems for causes of stillbirth and neonatal death in use 2009-2014. (DOCX 66 kb) [file 12884_2016_1071_MOESM11_ESM.docx]

## Additional file 11

### Kappa scores from reliability testing of classification systems for causes of stillbirth and neonatal death in use 2009-2014

| System | Overall agreement | Cause-specific agreement | Source |
| --- | --- | --- | --- |
| Cole 1986 | 35% |  | External [[1](#_ENREF_1)] |
|  | 85% and 92% | 58%-98% | External [[2](#_ENREF_2)] |
| de Galan-Roosen 2002 | 70% | 26% (trauma)-100% (maternal immune system pathology) | Internal [[3](#_ENREF_3)] |
| Flenady 2009-PSANZ-PDC | 63% |  | Internal [[1](#_ENREF_1)] |
|  |  | 92% (unexplained) | External [[4](#_ENREF_4)] |
| Froen 2009-Codac | 51%-82% for each of 21 pairs of raters |  | Internal [[5](#_ENREF_5)] |
|  | 65% |  | External [[1](#_ENREF_1)] |
| Gardosi 2005-ReCoDe | 51% |  | External [[1](#_ENREF_1)] |
| Korteweg 2006-Tulip | 81% | 46%-92% | Internal [[6](#_ENREF_6)] |
|  | 74% |  | External [[1](#_ENREF_1)] |
|  | 93% | 74%-96% | External [[2](#_ENREF_2)] |
| Nausheen 2013 |  | 9% (asphyxia)-72% | Internal [[7](#_ENREF_7)] |
| Ujwala 2012 | 65% |  | Internal [[8](#_ENREF_8)] |
| Van Diem 2010 | 87%-89% | 63%-94% | Internal [[2](#_ENREF_2)] |
| Varli 2008-Stockholm | 70% | 20% (other)-100% (twin to twin) | Internal [[9](#_ENREF_9)] |

1. Flenady V, Froen JF, Pinar H, Torabi R, Saastad E, Guyon G et al. An evaluation of classification systems for stillbirth. BMC Pregnancy Childbirth. 2009;9:24.

2. van Diem M, De Reu P, Eskes M, Brouwers H, Holleboom C, Slagter-Roukema T et al. National perinatal audit, a feasible initiative for the Netherlands!? A validation study. Acta Obstet Gynecol Scand. 2010;89(9):1168-73.

3. de Galan-Roosen AE, Kuijpers JC, van der Straaten PJ, Merkus JM. Fundamental classification of perinatal death. Validation of a new classification system of perinatal death. Eur J Obstet Gynecol Reprod Biol. 2002;103(1):30-6.

4. Hirst JE, Arbuckle SM, Do TM, Ha LT, Jeffery HE. Epidemiology of stillbirth and strategies for its prevention in Vietnam. Int J Gynaecol Obstet. 2010;110(2):109-13.

5. Froen JF, Pinar H, Flenady V, Bahrin S, Charles A, Chauke L et al. Causes of death and associated conditions (Codac) - a utilitarian approach to the classification of perinatal deaths. BMC Pregnancy Childbirth. 2009;9(22).

6. Korteweg FJ, Gordijn SJ, Timmer A, Erwich JJ, Bergman KA, Bouman K et al. The Tulip classification of perinatal mortality: introduction and multidisciplinary inter-rater agreement. BJOG. 2006;113(4):393-401. doi:10.1111/j.1471-0528.2006.00881.x.

7. Nausheen S, Soofi SB, Sadiq K, Habib A, Turab A, Memon Z et al. Validation of Verbal Autopsy Tool for Ascertaining the Causes of Stillbirth. PLoS One. 2013;8(10).

8. Ujwala B, Alcock G, More NS, Sushmita D, Wasundhara J, Osrin D. Stillbirths and newborn deaths in slum settlements in Mumbai, India: a prospective verbal autopsy study. BMC Pregnancy Childbirth. 2012;12(39).

9. Varli IH, Petersson K, Bottinga R, Bremme K, Hofsjo A, Holm M et al. The Stockholm classification of stillbirth. Acta Obstet Gynecol Scand. 2008;87(11):1202-12. doi:10.1080/00016340802460271.
