# Supplementary material for: Seeking order amidst chaos: a systematic review of classification systems for causes of stillbirth and neonatal death, 2009–2014
Source: BMC Pregnancy Childbirth. 2016 Oct 5;16:295. doi: 10.1186/s12884-016-1071-0 (PMC5053068; doi:10.1186/s12884-016-1071-0)
Supplement: Additional file 12: — Selected shortcomings of existing systems and rationale for development of new systems/modification of existing systems. (DOCX 44 kb) [file 12884_2016_1071_MOESM12_ESM.docx]

## Additional file 12

### Selected shortcomings of existing systems and rationale for development of new systems/modification of existing systems

| Shortcomings of existing systems | Rationale for development of new or modified systems |
| --- | --- |
| - Not comprehensive enough (e.g., insufficient placental subcategories) - Don’t address underlying causes or confuse them with other factors - Not holistic: focus on what, or when, or why, rather than all together; don't address mother, fetus and placenta together - If feasible for low-income countries, don’t accommodate complex systems - Lack definitions and guidelines - Do not accommodate recent knowledge on causation - Focus either on clinical or pathological data rather than both - Don't focus on autopsy - Have a high proportion of “unexplained” deaths - Either ignore fetal growth restriction or consider it to be causal - Address NND and SB separately - Address NND and SB together - Aren't hierarchical - Are hierarchical | - To add features (e.g. what, where and when; levels of probability; contributing factors) - To add missing categories - To increase comprehensiveness (include clinical, pathologic, histological, and/or autopsy findings) - To increase accuracy and produce actionable data - To reach new audiences (e.g. specific HIC, developing regions) - To enable identification of underlying causes…or not (one system deliberately does not seek underlying causes) - To provide consistency (uniform & clear definitions) - To reduce the number of “unexplained” deaths - To overcome other shortcomings (e.g., improve information management, be useful for all populations, relate conceptually to the ICD) |
